# miR1432‐*OsACOT* (Acyl‐CoA thioesterase) module determines grain yield via enhancing grain filling rate in rice

**DOI:** 10.1111/pbi.13009

**Published:** 2018-10-08

**Authors:** Ya‐Fan Zhao, Ting Peng, Hong‐Zheng Sun, Sachin Teotia, Hui‐Li Wen, Yan‐Xiu Du, Jing Zhang, Jun‐Zhou Li, Gui‐Liang Tang, Hong‐Wei Xue, Quan‐Zhi Zhao

**Affiliations:** ^1^ Collaborative Innovation Center of Henan Grain Crops Henan Agricultural University Zhengzhou China; ^2^ Key Laboratory of Rice Biology in Henan Province Henan Agricultural University Zhengzhou China; ^3^ Department of Biological Sciences and Biotechnology Research Center (BRC) Michigan Technological University Houghton MI USA; ^4^ National Key Laboratory of Plant Molecular Genetics CAS Center for Excellence in Molecular Plant Sciences Shanghai Institute of Plant Physiology and Ecology Chinese Academy of Sciences (CAS) Shanghai China

**Keywords:** rice, miR1432, grain filling, grain size, *OsACOT*

## Abstract

Rice grain filling rate contributes largely to grain productivity and accumulation of nutrients. MicroRNAs (miRNAs) are key regulators of development and physiology in plants and become a novel key target for engineering grain size and crop yield. However, there is little studies, so far, showing the miRNA regulation of grain filling and rice yield, in consequence. Here, we show that suppressed expression of rice miR1432 (STTM1432) significantly improves grain weight by enhancing grain filling rate and leads to an increase in overall grain yield up to 17.14% in a field trial. Molecular analysis identified rice Acyl‐CoA thioesterase (*OsACOT*), which is conserved with ACOT13 in other species, as a major target of miR1432 by cleavage. Moreover, overexpression of miR1432‐resistant form of *OsACOT* (OXmACOT) resembled the STTM1432 plants, that is, a large margin of an increase in grain weight up to 46.69% through improving the grain filling rate. Further study indicated that *OsACOT* was involved in biosynthesis of medium‐chain fatty acids. In addition, RNA‐seq based transcriptomic analyses of transgenic plants with altered expression of miR1432 demonstrated that downstream genes of miR1432‐regulated network are involved in fatty acid metabolism and phytohormones biosynthesis and also overlap with the enrichment analysis of co‐expressed genes of *OsACOT*, which is consistent with the increased levels of auxin and abscisic acid in STTM1432 and OXmACOT plants. Overall, miR1432‐*OsACOT* module plays an important role in grain filling in rice, illustrating its capacity for engineering yield improvement in crops.

## Introduction

Rice (*Oryza sativa* L.) is a staple food for more than half of the world's population and yield improvement is a major goal of breeders. Rice grain yield is a complex trait multiplicatively determined by its three component traits: number of panicles, number of grains per panicle and grain weight. Grain weight, as the most important factor determining yield, is largely determined by the filling rate and duration of the filling period of rice. More importantly, rate of grain filling contributes largely to grain productivity (Zhou *et al*., 2013) and mainly supplies the endosperm development and accumulation of nutrients. Genetics studies have identified many grain weight‐related QTLs (Si *et al*., 2016; Zuo and Li, 2014), however, the knowledge about regulatory networks controlling grain weight and rice yield remains limited due to the involvement of various processes and regulatory factors which are regulated post‐transcriptionally or post‐translationally. Therefore, new gene resources that could control the complex traits are still urgently desired for rice cultivation.

MicroRNAs (miRNAs), a class of 19–24 nt abundant small non‐coding RNAs, are important regulators in both plants and animals, regulating the expression of their target genes (Achkar *et al*., 2016; Bartel, 2004, 2009). In the past few years, studies have demonstrated the crucial roles of miRNAs in various aspects of plant growth and development including organ morphogenesis, hormone function, signal transduction and response to environmental stimuli. More importantly, there are also reports illustrating the involvement of miRNAs in regulating agronomic traits (Tang and Chu, 2017; Zhang *et al*., 2017a), especially seed development (Peng *et al*., 2014; Xue *et al*., 2009; Yi *et al*., 2013). Recently, a list of studies in rice reported that altering the expression of a single miRNA could significantly change crop yield, including grain shape and size by regulating grain‐related characteristics. Specifically, miR156 could define panicle branching by down‐regulating its target *OsSPL13, OsSPL14* and *OsSPL16* to consequently improve grain productivity (Jiao *et al*., 2010; Miura *et al*., 2010). Blocking of miR396 resulted in different inflorescence architecture and hence increased grain size and rice yield (Gao *et al*., 2010; Hu *et al*., 2015; Li *et al*., 2016). Overexpressing miR397 increased the overall grain yield up to 25% by enlarging grain size and promoting panicle branching (Zhang *et al*., 2013). OsmiR1848 regulates *OsCYP51C* expression and mediates BR biosynthesis to modulate leaf angle, grain size and seed quality (Xia *et al*., 2015). Most recently, it has been reported that miR408 could regulate grain yield and photosynthesis via a phytocyanin protein, OsUCL8 (Zhang *et al*., 2017b). Manipulation of miR398 can increase panicle length, grain number and grain size (Zhang *et al*., 2017a) and suppressing expression of miR159 affects multiple traits of rice including grain shape by up‐regulating its targets *OsGAMYB* and *OsGAMYBL1* (Zhao *et al*., 2017). Overexpression of miR164b‐resistant target, *OsNAC2,* could make better plant architecture, longer panicles, more grain number and yield (Jiang *et al*., 2018). However, so far there is little information showing the effect of miRNA on rice yield through affecting grain filling by negatively regulating its target.

Therefore, identification of new regulatory networks and studies of the relevant mechanisms contributed by miRNAs on grain filling will surely help to understand the complex control of grain weight, thereby improving the grain yield and quality. Our previous studies have identified the differentially expressed miRNAs between rice superior and inferior grains during filling (Peng *et al*., 2011, 2014), which provides informative clue to study the role of miRNAs in filling rate regulation. Among the differentially expressed miRNAs, miR1432 shows higher expression in inferior grains than that in superior grains during seed development. Moreover, the expression levels of miR1432 during grain filling increases gradually and show a relative opposite correlation with grain filling rate, suggesting a potential role of miR1432 in grain size regulation and grain filling process. Thus, miR1432 was selected for detailed analysis to explore its possible function in seed development. In present study, we focus on studying the role of miR1432 in rice grain filling and seed size determination, and prediction and validation of its downstream target. Preliminary analysis of the target's function and application of miR1432‐target module in rice yield improvement suggest that rice miR1432 is a promising candidate gene in breeding for crop improvement.

## Results

### miR1432 is highly expressed in developing rice seeds

**Figure 1:**
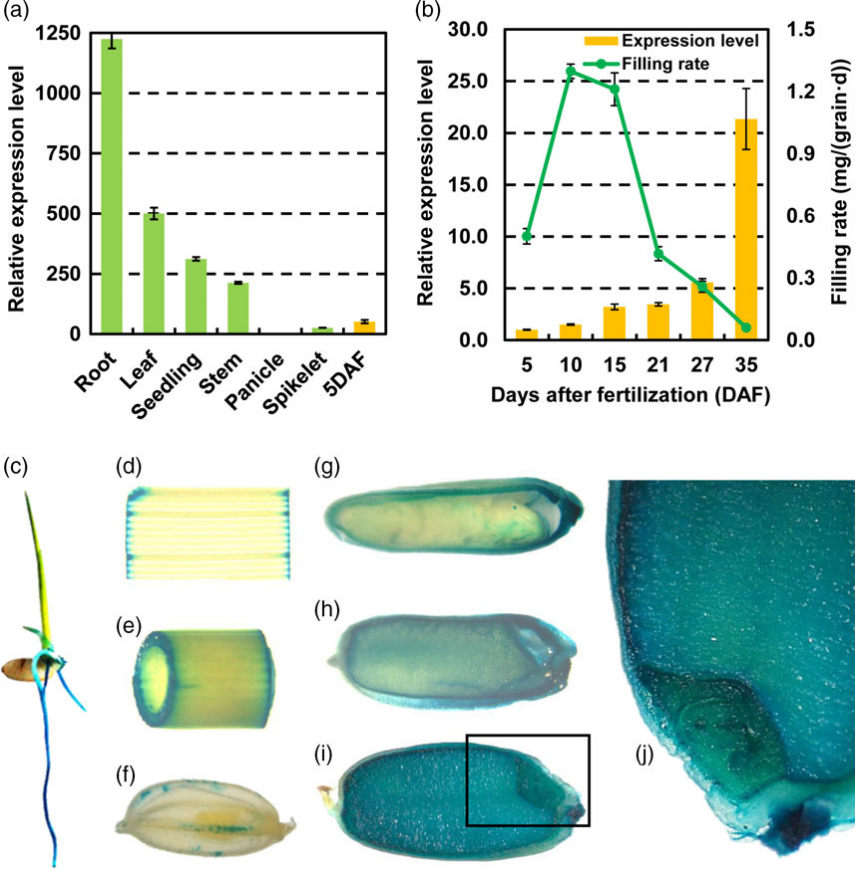
Expression pattern analysis of rice miR1432 showed a negative correlation with grain filling rate. (a) Expression analysis of rice miR1432 in vegetative and reproductive organs till 5 days after fertilization (DAF) by stem‐loop qPCR; (b) Expression of rice miR1432 and grain filling rate in *Nipponbare* plants during grain development. Experiments were repeated in three independent biological samples and error bars indicate standard deviations of three biological replicates. (c‐i) Spatial expression of miR1432 by GUS staining. (c) seedling; (d) leaf; (e) stem; (f) spikelet; (g‐i) developing grains at 5 DAF; (g) 15 DAF (h) and 30DAF (i) respectively; (j) magnified view of the boxed area in (i) (30 DAF grain); analysis was repeated in three independent biological samples and representative images were shown.

In order to investigate the spatial expression patterns of miR1432 during rice development, qRT‐PCR and β‐glucuronidase (GUS) activity assays were used to analyse miR1432 expression in various organs of wild type (WT) plants. The expression level of miR1432 varied among different tissues and developmental stages (Figure 1). It was found that miR1432 is preferentially highly expressed in root (Figures 1a,c), seedling (Figure 1a), stem (Figures 1a,e), and developing seeds (Figures 1b,g–j), whereas it is lowly expressed in other tissues including panicle (Figure 1a) and spikelets (Figures 1a,f). Specifically, the transcriptional level of miR1432 increased along with the seed development, which was negatively correlated with the grain filling rate (Figures 1b,g–i), indicating that miR1432 may have roles in controlling rice seed development.

### miR1432 negatively regulates grain size in rice

**Figure 2:**
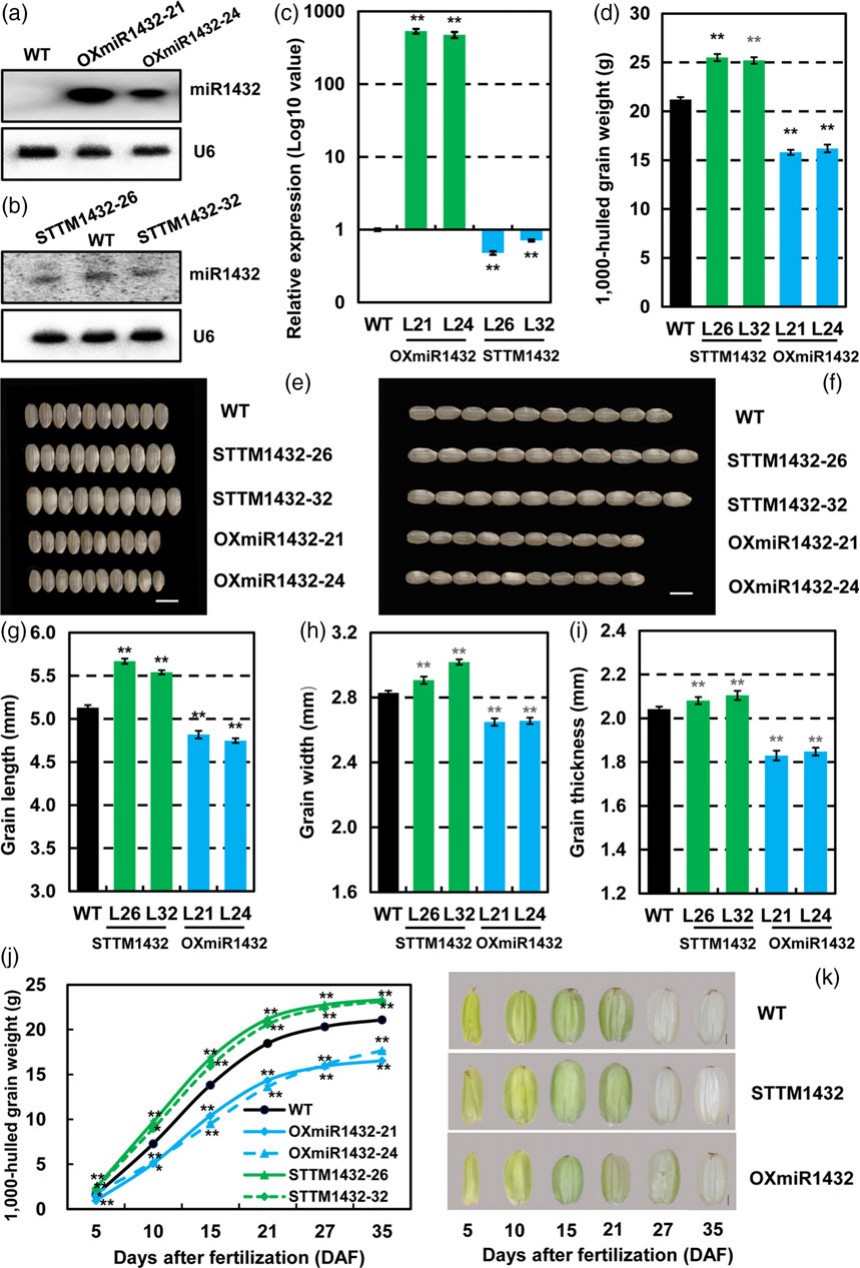
Rice miR1432 negatively regulates grain filling (in the year 2017‐Zhengzhou). Northern blot analysis of enhanced (a) or suppressed (b) expressions of rice miR1432 in OXmiR1432 or STTM1432 transgenic plants respectively; (c) Validation of increased or decreased expressions of rice miR1432 in OXmiR1432 or STTM1432 transgenic plants respectively, by stem‐loop qRT‐PCR; (d) Measurement of the 1,000‐hulled grain weight of *Nipponbare* (WT), STTM1432, and OXmiR1432 transgenic plants; (e‐f) Phenotypic observation of grain size of *Nipponbare* (WT), STTM1432 and OXmiR1432 transgenic plants. Scale bars, 5 mm; (g‐i) Detailed analysis of grain traits including grain length (g), width (h), and thickness (i) of *Nipponbare* (WT) and miR1432 transgenic plants; (j) Measurements of grain filling rate in *Nipponbare* (WT), STTM1432 and OXmiR1432 transgenic plants; experiments were repeated three times and data are presented as mean ± SD (*n *=* *1000 grains); statistical analysis was performed by Student's *t*‐test (**P <* *0.01; *P <* *0.05). (k) Morphologies of seeds of *Nipponbare* (WT), STTM1432 and OXmiR1432 transgenic plants at different stage of grain filling, Scale bar, 1 mm.

To evaluate the effect of miR1432 on rice grain development, its expression in *Nipponbare* endosperm was either increased (OXmiR1432) or suppressed by STTM (STTM1432), driven by an endosperm‐specific promoter Gt13a respectively. To detect whether expressions of miR1432 changed or not, northern blot and qRT‐PCR were used to analyse its expression level in 10 DAF endosperm. As expected, expressions of miR1432 increased in OXmiR1432 transgenic plants (Figures 2a,c), whereas expression of miR1432 was suppressed effectively in STTM1432 transgenic lines compared with that of WT (Figures 2b,c).

Phenotypic observation of the positive transgenic lines in 3 years of field experiments showed that altered miR1432 expression results in the changed grain size, i.e. bigger or smaller seeds under suppressed or enhanced expressions of miR1432 respectively (Figures 2e–f, S1a,b, S2). Detailed measurement of grain‐related traits revealed the increased 1,000‐hulled grain weight 18.88%–20.28% (*P *<* *0.01) (Figures 2d, S1c), grain length 8.03%–10.54% (*P *<* *0.01) (Figures 1g, S1e), width 4.76%–6.72% (*P *<* *0.01) (Figures 2h, S1f) and thickness 1.96%–3.11% (*P *<* *0.01) (Figures 2i, S1g) of STTM1432 seeds without any notable difference in aspects of main panicle features, such as length of main panicle, numbers of primary and secondary branches, effective grain numbers and seed setting rate per panicle (Tables 1 and S1, Figure S2a). On the contrary, increased expression of miR1432 (OXmiR1432) results in the seeds with decreased length, width and thickness, and hence reduced 1,000‐hulled grain weight (Figures 2d–f, S1a–c, S2). In brief, grain length, grain width and grain thickness significantly reduced 8.03%–10.54% (*P *<* *0.01) (Figures 2g, S1e), 6.10%–6.35% (*P *<* *0.01) (Figure 2h, S1f), and 9.48%–10.34% (*P *<* *0.01) (Figures 2i, S1g) in OXmiR1432 transgenic plants when compared with wild‐type plants respectively. Consequently, 1,000‐hulled grain weight decreased 23.58%–25.47% (*P *<* *0.01) (Figures 2d, S1c), indicating a negative effect of miR1432 in seed development and yield. In addition, the actual yield of the transgenic plants under the altered expressions of miR1432 was measured using field experiments. Overall, the yield in STTM1432 plants increased by 13.20%–17.14% (*P *<* *0.01), while in OXmiR1432 plants decreased by 17.94%–29.12% (*P *<* *0.01) (Tables 1 and S1).

**Table 1:** Grain yield and associated components of transgenic plants with altered expressions of miR1432 and its target in field trials

Characters	WT	OXmiR1432		STTM1432		OXmACOT	
		L21	L24	L26	L32	L1	L3
Tiller numbers	17.67 ± 0.50	16.17 ± 0.57	16.20 ± 0.47	17.20 ± 0.52	17.27 ± 0.47	16.93 ± 0.57	16.67 ± 0.47
Spikelet number	128.15 ± 2.02	126.53 ± 1.58	130.5 ± 2.47	127.93 ± 2.02	120.13 ± 2.14	135.95 ± 1.39	132.32 ± 1.62
Seed setting rate (%)	92.35 ± 0.39	91.64 ± 0.56	92.22 ± 1.12	90.84 ± 0.46	93.26 ± 0.39	91.02 ± 0.38	91.73 ± 0.30
Length of panicle (cm)	21.58 ± 0.33	20.03 ± 0.15	20.57 ± 0.16	22.44 ± 0.41	20.71 ± 0.21	21.22 ± 0.15	22.09 ± 0.20
Primary branch numbers	12.03 ± 0.11	12.17 ± 0.14	12.82 ± 0.20	12.60 ± 0.16	12.05 ± 0.13	11.83 ± 0.14	12.42 ± 0.19
Secondary branch numbers	20.23 ± 0.59	20.87 ± 0.49	21.87 ± 0.63	19.05 ± 0.55	19.05 ± 0.63	19.42 ± 0.63	20.77 ± 0.81
1,000‐hulled grain weight (g)	21.20 ± 0.26	15.80 ± 0.26	16.20 ± 0.39	25.50 ± 0.36	25.20 ± 0.33	29.45 ± 0.28	31.10 ± 0.33
Grain yield per plant (g)	44.30 ± 3.20	32.50 ± 3.30	35.70 ± 4.10	52.10 ± 4.70	50.90 ± 4.50	57.70 ± 5.40	58.90 ± 2.30
Yield increase (%)	–	−26.64	−19.41	17.61	14.90	30.20	32.90
Grain yield (kg per plot)	1.55 ± 0.01	1.10 ± 0.11**	1.27 ± 0.06**	1.81 ± 0.13**	1.75 ± 0.07**	–	–
Yield increase (%)	–	−29.12	−17.94	17.14	13.20	–	–

Values shown are the mean ± SD (*n *=* *60 panicles, *n *=* *10 plants, *n *=* *3 plots). Significant differences were identified using Student's *t*‐test. **P <* *0.01.

### miR1432 controls rice grain size through altering grain filling rate

Considering the concomitant increase in the expression of miR1432 with seed maturation and importance of grain filling in contributing to seed size and yield, the seed filling rate in plants with altered miR1432 expression was firstly examined. Results showed that compared with WT, the grain filling rate of STTM1432 was significantly increased, while that of OXmiR1432 showed significant decrease along with seed development (Figures 2j–k, S1d). This is consistent with the altered miR1432 expression in endosperm and increased or decreased grain weight of STTM1432 or OXmiR1432 seeds respectively, which suggests the possible role of miR1432 in filling regulation. To further detect the filling characters, detailed parameters related to filling, such as initial filling potential, maximum and mean filling rate, date reaching maximum filling rate, days of active grain filling were investigated and fitted according to Richards equation (Benjamini and Hochberg, 1995). Among these parameters, maximum filling rate increased or decreased 0.25 mg/(grain·day) (*P *<* *0.01) or 0.56 mg/(grain·day) (*P *<* *0.01) and mean filling rate increased or decreased 0.14 mg/(grain·day) (*P *<* *0.01) or 0.32 mg/(grain·day) (*P *<* *0.01) in STTM1432 and OXmiR1432 transgenic plants respectively (Tables 2 and S2). These results confirmed miR1432 held an important role in rice grain filling regulation and repression of miR1432 can increase grain weight by enhancing the grain filling rate.

**Table 2:** The grain filling parameters for wild‐type and different transgenic plants of miR1432 and OXmACOT

Parameters	WT	OXmiR1432		STTM1432		OXmACOT	
		L21	L24	L26	L32	L1	L3
GR0	3.48 ± 0.34	2.09 ± 0.31	2.65 ± 0.30	4.69 ± 0.53	3.93 ± 0.17	2.46 ± 0.56	1.89 ± 0.34
Vamax (mg/(grain·day))	1.40 ± 0.01	1.09 ± 0.05**	0.84 ± 0.04**	1.65 ± 0.02**	1.55 ± 0.05**	1.72 ± 0.02**	1.73 ± 0.03**
Va (mg/(grain·day))	0.91 ± 0.01	0.76 ± 0.03**	0.59 ± 0.03**	1.02 ± 0.02**	1.05 ± 0.03**	1.17 ± 0.01**	1.18 ± 0.04**
tmax (day)	10.54 ± 0.41	11.21 ± 0.19	12.03 ± 0.32	9.46 ± 0.56	9.88 ± 0.54	10.37 ± 0.38	10.39 ± 0.36
Active period (day)	22.23 ± 0.33	21.94 ± 0.35	21.57 ± 0.49	22.63 ± 0.61	22.20 ± 0.21	24.13 ± 0.34	24.00 ± 0.67

GR0: Initial filling potential; Vam: Maximum filling rate; Va: Mean filling rate; tmax: Date when reaches maximum filling rate; Active period: Days of active grain filling. Values shown are the mean ± SD (*n *=* *60). Significant differences were identified using Student's *t*‐test. **P <* *0.01.

### *OsACOT* is targeted by miR1432 through cleavage

It is well known that plant miRNAs have near‐perfect pairing with their targets and function through cleaving them directly, or in some cases, by their translational repression. Therefore, identifying miRNAs target genes is important to understand their specific contributions. To identify the molecular mechanism of miR1432 regulating grain size, potential targets of miR1432 were first computationally predicted using psRNATarget (http://plantgrn.noble.org/psRNATarget/), a miRNA target prediction online tool (Dai *et al*., 2018). In total, 16 potential targets of miR1432 were predicted with relatively strict parameters, and all of them belong to different protein families (Table S4). In general, the expression of miRNAs shows a negatively correlation with their real targets. RNA‐seq results showed only *LOC_Os04g35590* to be highly expressed in STTM1432 and lowly expressed in OXmiR1432 transgenic plants (Table S5). Therefore, it was speculated as the most prospective target of miR1432.

**Figure 3:**
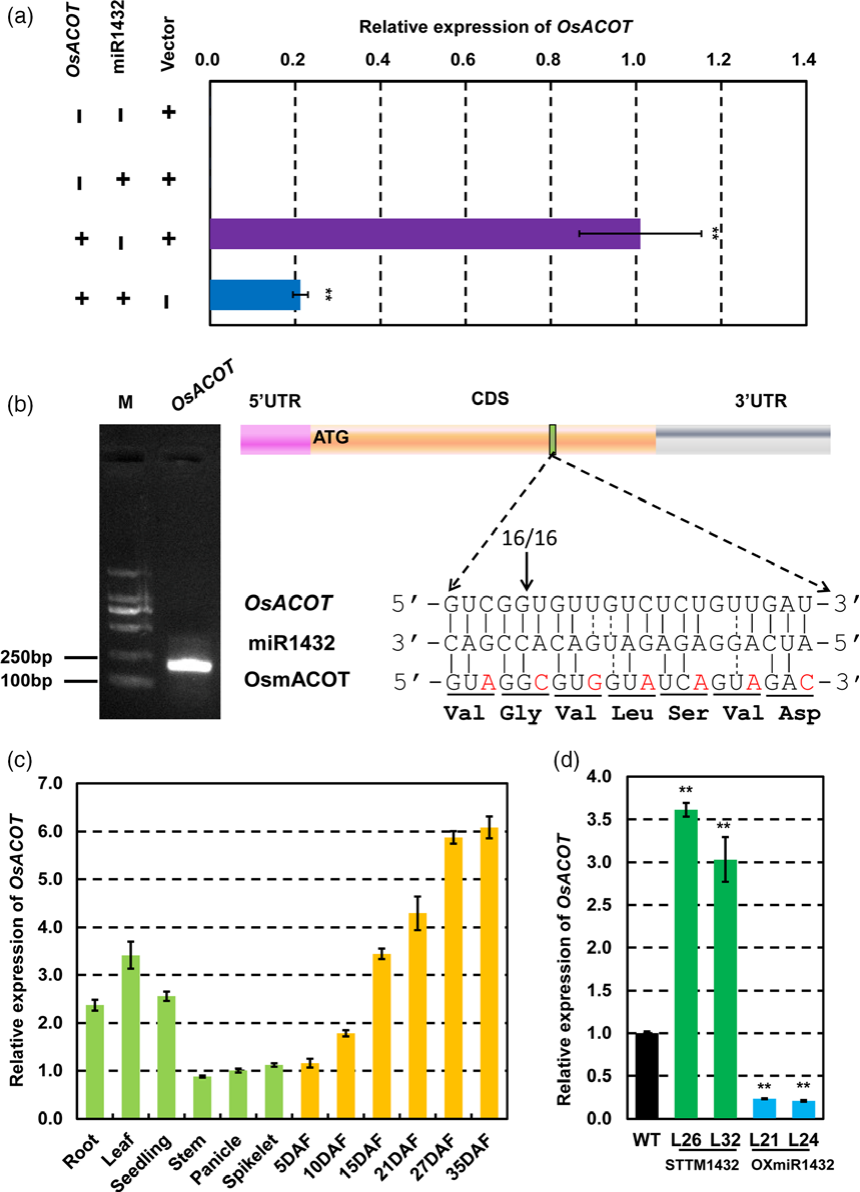
Rice miR1432 negatively regulates the expression of *OsACOT*. (a) *OsACOT* was shown to be cleaved by miR1432 using *Nicotiana. benthamiana*‐based *in vitro* analysis; (b) Rice miR1432 cleavage site in *OsACOT* mRNA was confirmed by RNA ligase–mediated 5′‐RACE; (c) Expression pattern of *OsACOT* at vegetative and reproductive stages was analysed by quantitative real‐time PCR (qPCR); (d) Suppressed or enhanced expressions of *OsACOT* in rice endosperms at 10 DAF (days after fertilization) of *Nipponbare* (WT), STTM1432 and OXmiR1432 transgenic plants; experiments were repeated three times and data are presented as mean ± SD (*n *=* *3); statistical analysis was performed by Student's *t*‐test (**P <* *0.01).

To further validate whether *LOC_Os04g35590* is the real target of miR1432, agroinfilitration of *Nicotiana benthamiana* leaves, as a transient expression method, was used to validate the interaction between miR1432 and its putative targets *in vitro*. As expected, transcript expression of *LOC_Os04g35590* decreased sharply when fused with the precursor of miR1432 (Figure 3a). To further validate the target of miR1432, we mapped the miR1432‐directed cleavage sites in *OsACOT* using RNA ligase‐mediated rapid amplification of cDNA ends (RLM‐RACE). The results showed that cleavage occurred between the 16th and 17th base pair of the miR1432 target site (Figure 3b), indicating that *OsACOT* can be precisely cleaved *in vivo* by miR1432. Furthermore, we investigated the spatial expression pattern of *OsACOT* in various rice tissues using qRT‐PCR analysis (Figure 3c). It revealed that *OsACOT* was mainly expressed in roots and developing seeds, which was similar to the spatial expression pattern of miR1432. The expression levels of *OsACOT* were negatively correlated with those of miR1432 in STTM1432 or OXmiR1432 transgenic lines (Figure 3d). All these results indicate that *OsACOT* is an authentic target gene of miR1432 and its expression is negatively regulated by miR1432‐directed cleavage.

### Overexpression of miR1432‐resistant *OsACOT* phenocopies STTM1432 plants

**Figure 4:**
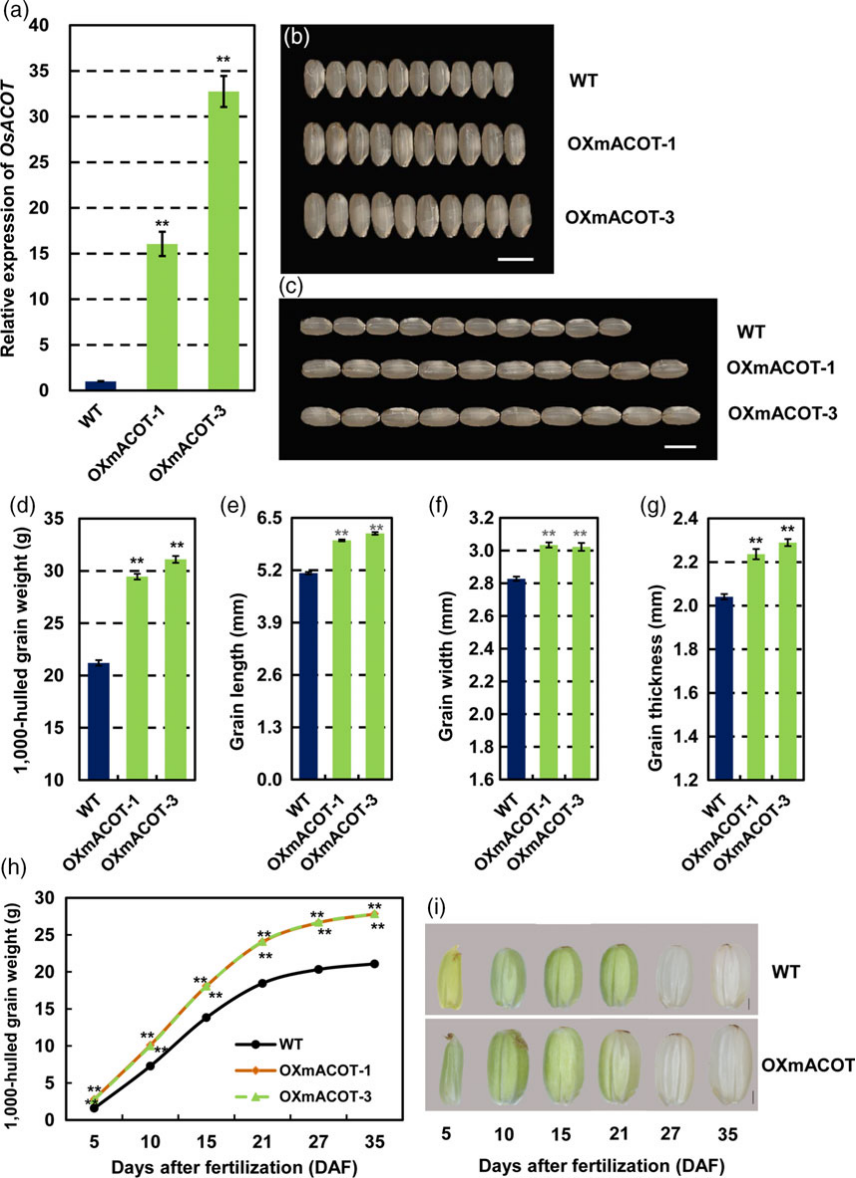
*OsACOT* positively regulates rice grain filling (in the year 2017‐Zhengzhou). (a) Expressions levels of *OsACOT* in endosperm at 10 DAF (days after fertilization) of *Nipponbare* (WT) and OXmACOT plants; (b‐c) Phenotypic observation of *Nipponbare* (WT) and OXmACOT grains; Scale bars, 5 mm; (d‐g) Measurements of 1,000‐hulled grain weight (d) and detailed analysis of grain traits including grain length (e), width (f) and thickness (g); (h) Measurements of grain weight of *Nipponbare* (WT) and OXmACOT transgenic plants during grain filling; (i) Morphologies of seeds of *Nipponbare* (WT) and OXmACOT transgenic plants at different stage of grain filling, Scale bar, 1 mm; experiments were repeated three times and data are presented as mean ± SD (*n* = 1000 grains); statistical analysis was performed by Student's *t*‐test (**P <* *0.01).

To further check whether miR1432‐mediated negative regulation of *OsACOT* affects grain size and filling rate, we hypothesized that higher expression of *OsACOT* in the endosperm of rice would lead to an increase in grain productivity. Therefore, we generated transgenic plants (OXmACOT) expressing an miR1432‐cleavage resistant *OsACOT* gene (m*ACOT*) that contained seven mismatches to the site targeted by miR1432 without any amino acid changes with Gt13a endosperm‐specific expression promoter (Figure 3b). qRT‐PCR analysis of *OsACOT* mRNA expression in these transgenic lines indicated significantly increased level of *OsACOT* mRNA in the OXmACOT transgenic lines (Figure 4a). To further explore the relationship between the dosage of the *OsACOT* mRNA and the grain weight, expression of *OsACOT* in OXmACOT plants with varying grain weights was examined. As expected, a significant positive correlation was observed between the *OsACOT* expression level and the 1000‐hulled grain weight (Figure S5a–c). Moreover, consistent with that of STTM1432, grain size of OXmACOT was significantly increased compared to WT in the two sites’ field experiments (Figures 4b,c, S3a,b, S4) and the grain length, grain width, and grain thickness increased 15.87%–19.24% (*P *<* *0.01), 6.89%–7.30% (*P *<* *0.01) and 9.55%–12.18% (*P *<* *0.01) respectively. Overall, 1,000‐hulled grain weight significantly increased 38.92%–46.70% (*P *<* *0.01) (Figures 4d–g, S3c, e–g). Further detailed analysis of grain filling parameters indicated both mean and maximum filling rate of OXmACOT transgenic plants to be significantly higher than those of WT (Figures 4h–i, S3d, Tables 2 and S3). Specifically, maximum filling rate and mean filling rate increased 0.33 mg/(grain·day) (*P *<* *0.01) and 0.27 mg/(grain·day) (*P *<* *0.01) respectively. All these evidences further support the hypothesis that STTM1432 increased grain filling through down‐regulation of miR1432, which resulted into decreased miR1432‐guided cleavage of the *OsACOT* mRNA. In addition, we tried to generate the mutant of *OsACOT* by means of CRISPR/Cas9 technology. However, the resistant calli could not differentiate into seedlings in two independent transformations. Lethality under *OsACOT* deficiency also indicates the role of *OsACOT* in regulating key plant functions.

### *OsACOT* regulates fatty acid metabolism

**Figure 5:**
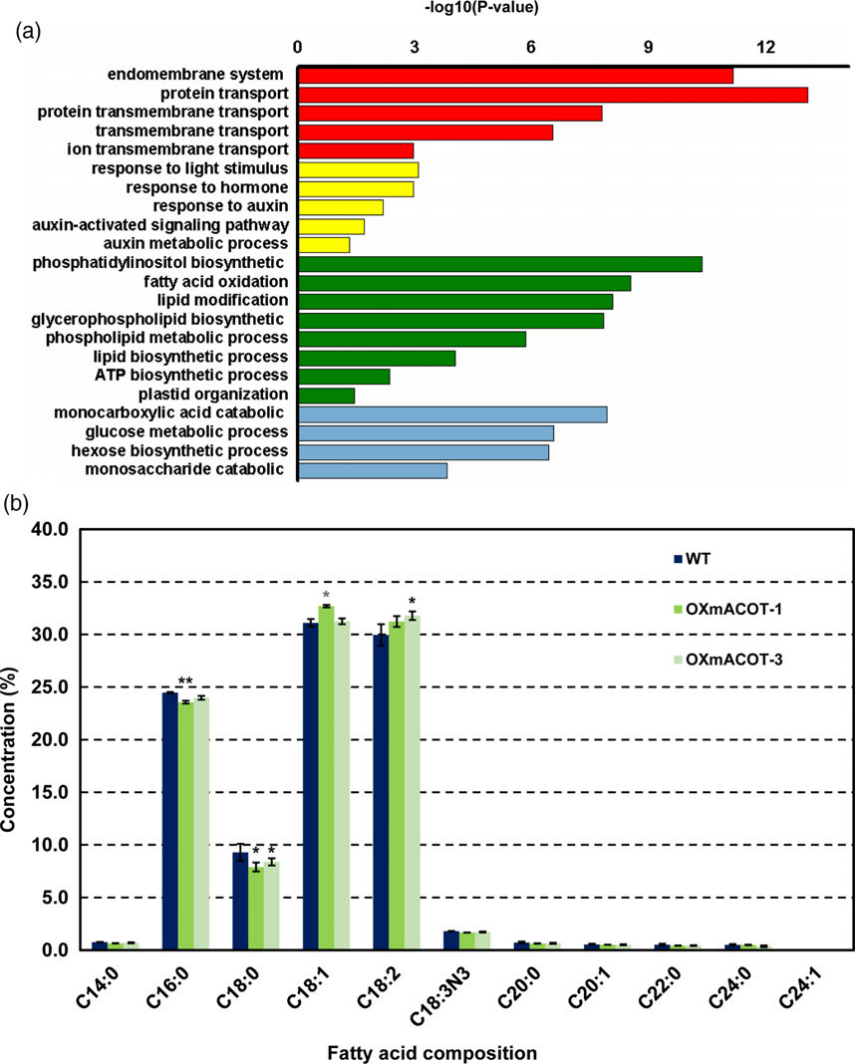
*OsACOT* regulates fatty acid composition in rice. (a) GO enrichment analysis of *OsACOT* co‐expressed genes. Hypergeometric test was used with subsequent Benjamini and Hochberg false discovery rate corrections. Only GO terms with a corrected *P*‐value <0.05 and at least 5 annotated genes were retained. Length of bars represents negative logarithm (base 10) of the corrected *P*‐value. (b) Fatty acid compositions of wild type and OXmACOT transgenic plants. Brown grains without embryos were used for fatty acid composition analysis; experiments were repeated three times and data are shown as means ± SD; statistical analysis was performed by Student's *t*‐test (**P <* *0.01; *P <* *0.05).

To further clarify the function of *OsACOT* in rice seed development, we developed a coexpression network of *OsACOT* from rice endosperm development chip data. Gene‐clustering analysis indicated that co‐expressed genes of *OsACOT* are notably enriched in the pathways related to lipid biosynthesis and endomembrane system organization, hormone signalling and so on (Figures 5a, S6), suggesting a possible role of *OsACOT* in regulating these pathways. In addition, the *OsACOT* co‐expressed genes were up‐regulated in STTM1432 and OXmACOT transgenic plants but down‐regulated in OXmiR1432 plants (Figure S7). Therefore, fatty acids in brown rice of wild type and OXmACOT transgenic plants were identified by GC‐MS. In total, eleven fatty acids and their distribution was detected (Figure 5b). Significant differences were observed for 16:0 (palmitic acid), 18:0 (stearic acid), 18:1 (oleic acid) and 18:2 (linoleic acid) fatty acid contents between OXmACOT transgenic plants and wild type at *P *<* *0.05 level. Interestingly, contents of 16:0 and 18:0 fatty acids were lower and those of 18:1 and 18:2 fatty acids were higher in OXmACOT transgenic plants compared to wild‐type grains. All these results strongly suggested that *OsACOT* might be a key enzyme in fatty acid desaturation and elongation pathway, especially from fatty acid 16:0 to 18:2.

### Changed ABA and IAA levels in STTMmiR1432 and OXmACOT transgenic plants

To further explore the role of miR1432 in rice seed development, differentially expressed genes (DEGs) were studied between STTM1432/OXmiR1432 and wild type developing grains by high throughput RNA‐sequencing. The genes which were differentially expressed at least 1.5‐fold were identified and subjected to further analysis. Enrichment analysis showed that DEGs are mainly involved in endomembrane organization, fatty acid metabolism, lipid biosynthesis, sugar and starch metabolism. Most importantly, biological processes involved in hormone signaling such as abscisic acid (ABA) and auxin (IAA) were also clustered (Figure S8a). The results were further confirmed by qRT‐PCR in miR1432 and OXmACOT transgenic plants (Figure S8b,c).

**Figure 6:**
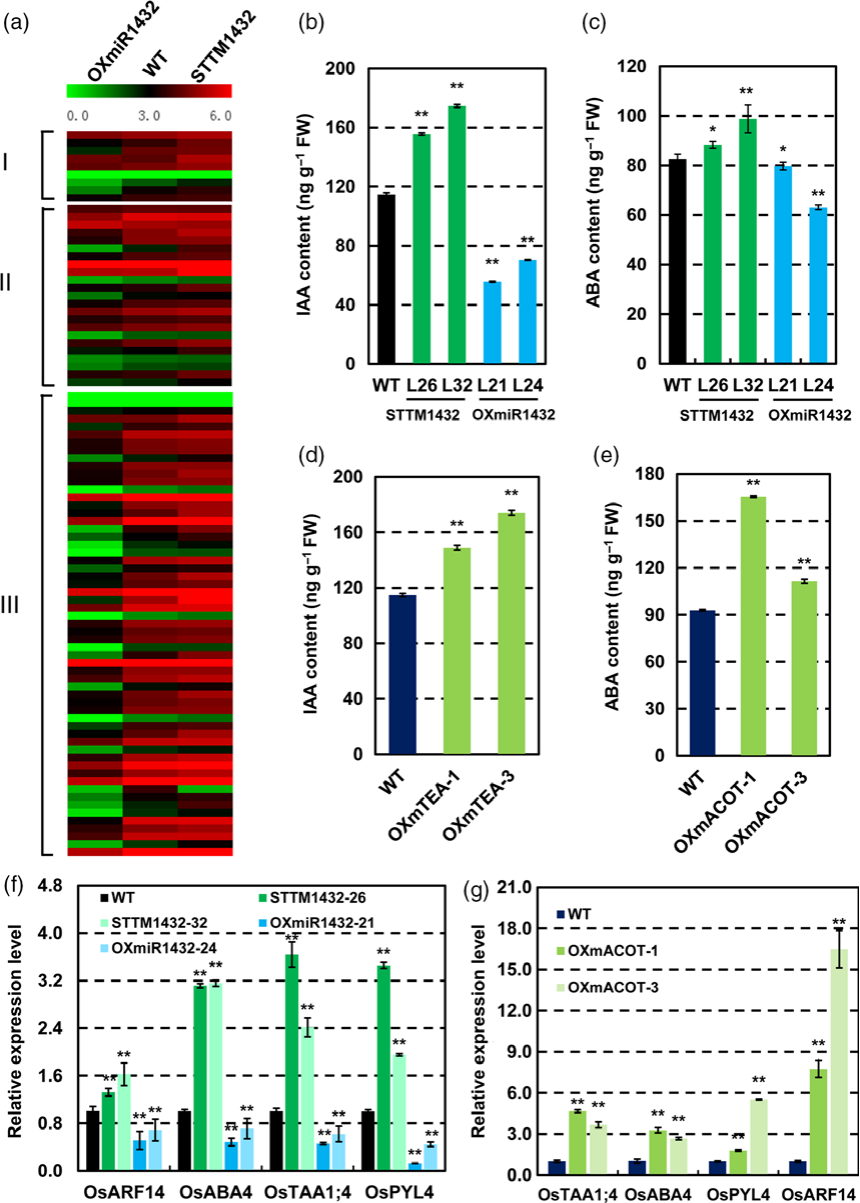
Rice miR1432‐*OsACOT* module is involved in IAA and ABA homeostasis. (a) Differentially expressed genes involved in IAA and ABA biosynthesis (I), IAA and ABA signal transduction (II), and their responsiveness (III), in *Nipponbare* (WT), STTM1432 and OXmiR1432 transgenic plants, identified by high throughput RNA‐sequencing analysis; (b‐c) Contents of IAA (b) and ABA (c) in *Nipponbare* (WT), STTM1432 and OXmiR1432 transgenic plants assayed by ESI‐HPLC‐MS/MS; (d‐e); Contents of IAA (d) and ABA (e) in *Nipponbare* (WT) and OXmACOT plants measured by ESI‐HPLC‐MS/MS; (f‐g) qRT‐PCR expression analysis of genes involved in biosynthesis and signaling of IAA and ABA in STTM1432 plants and OXmiR1432 plants (f), and were validated in OXmACOT transgenic lines by qRT‐PCR (g); Rice endosperms at 10 DAF (days after fertilization) were used for analysis; experiments were repeated three times and data are shown as means ± SD; statistical analysis was performed by Student's *t*‐test (**P <* *0.01; *P <* *0.05).

Interestingly, consistent with the idea that IAA and ABA are the positive regulators in grain filling and yield (Tan *et al*., 2009; Yang *et al*., 2006), there were nine DEGs, such as *OsTAA1* and *OsABA4* involved in IAA and ABA biosynthesis, and several DEGs, including *OsARF14*,* OsPYL/RCAR4* and series of DEGs related to IAA and ABA response among DEGs of STTM1432 transgenic plants and OXmiR1432 transgenic plants (Figure 6a). In addition, expressions of auxin efflux carriers, such as *OsPIN2*,* OsPIN9* and starch synthesis genes *OsBT1‐2*,* OsSSIIc* were found among the DEGs as well (Figure S8a) which were further confirmed by qRT‐PCR (Figure S8b,c). Furthermore, genes related to ABA synthesis and signaling were up‐regulated in STTM1432 and OXmACOT plants (Figure 6f,g). Specifically, expressions of genes related to IAA and ABA synthesis and signalling increased in STTM1432 and OXmACOT transgenic plants and decreased in OXmiR1432 transgenic plants. Therefore, we speculated that rice miR1432‐*OsACOT* module increases grain filling through activating the biosynthesis and responsiveness of IAA and ABA. In fact, endogenous IAA and ABA in endosperm at 10 DAF of WT and different transgenic plants were measured. Compared with WT, the contents of IAA and ABA were higher in the STTM1432 and OXmACOT transgenic plants, and lower in OXmiR1432 plants (Figure 6b–e). These results strongly suggested that miR1432‐*OsACOT* module regulates grain filling through modulating IAA and ABA biosynthesis and signalling.

## Discussion

Characterization of the roles that miRNAs play in the grain size and yield is an active field of research. Previous study reported that miR1432 was one of the miRNAs that was differentially expressed during grain filling between superior and inferior grains (Peng *et al*., 2014). In present study, we demonstrated a novel approach to enhance grain filling rate by manipulating miRNA expression. We found that altered expression of miR1432 in rice endosperm can significantly change grain filling, which in turn affect grain size and yield. Notably, a newly identified gene, *OsACOT*, which plays an important role in biosynthesis of fatty acid, is validated as the direct target gene of miR1432. Increased expression of miR1432‐resistant version of *OsACOT* (OXmACOT) results in the increased grain size and filling rate compared to those of WT plants. More importantly, field performance indicates that STTM1432 and OXmACOT transgenic plants can significantly increase plot yield by 17.14% and per plant yield by 32.90% respectively. These results suggest that miR1432‐*OsACOT* module play important roles in regulating rice yield by enhancing grain filling in rice.

### miR1432 regulates grain filling by promoting transport ability of endomembrane system through *OsACOT*

In present study, we found that an over‐expression of *OsACOT*, the direct target of miR1432, generated faster grain filling rate and enlarged grains. This finding suggested a positive role of *OsACOT* in grain size regulation. However, there are no published reports on the relationship of function between *OsACOT* and grain filling in rice. Furthermore, we searched for the orthologous genes of *OsACOT* among different species including model plant *Arabidopsis* (Figure S9). The orthologous genes in other species and KEGG (Kyoto Encyclopedia of Gene and Genomes) definition indicated that *OsACOT* might be the 13th member of acyl‐CoA thioesterase super family and is responsible for fatty acid and lipid biosynthesis (Figure S9). The altered compositions of fatty acids, especially C:16 to C:18, in brown rice of OXmACOT transgenic plants (Figure 5b), suggested the role of *OsACOT* in controlling lipid and fatty acid metabolism. Together with the idea that ACOT1/2/4 and ACOT7 in acyl‐CoA thioesterase super family play a certain role in long chain fatty acid elongation (Ellis *et al*., 2013), it is not difficult to speculate that *OsACOT* probably took part in the lipid metabolism of fatty acids, C:16 to C:18.

Lipid biosynthesis and metabolism is essential for endomembrane system organization. Numerous studies have demonstrated that plant endomembrane system is of vital importance for intracellular protein processing, lipid modification and transport, crucial for plant development and signal transduction (Boutte and Moreau, 2014; Debono *et al*., 2009; Ding *et al*., 2014; Morita and Shimada, 2014; Surpin and Raikhel, 2004). Especially, endomembrane system such as Golgi in developing endosperm cells is responsible for transportation of nutrients including storage protein to protein storage vacuole (Liu *et al*., 2013). Moreover, the defects of endomembrane would bring out abnormal starch structure and decreased grain filling (Wang *et al*., 2010). It is also reported that auxin transporters of the PIN‐FORMED (PIN) family localize asymmetrically at the plasma membrane (PM) and mediate directional intercellular auxin transport (Tanaka *et al*., 2006; Vanneste and Friml, 2009). It has been reported that PM‐located PIN proteins play important role in regulating cytosolic auxin concentration (Scherer, 2011). Combined with the results of co‐expressed genes of *OsACOT* and their overlap with the highly expressed genes involved in endomembrane system organization, auxin transport and starch synthesis in OXmACOT transgenic plants, it is very likely to accept that increased expression of *OsACOT* favours nutrients and auxin transport through the organization of endomembrane system, which in turn improves grain filling to enlarge grain size.

### miR1432‐*OsACOT* module mediates lipid and auxin signal transduction

Lipids are essential structural constituents of membranes and some also have important cell signaling roles, such as those involved in abscisic acid, salicylic acid and auxin pathways (Janda *et al*., 2013). *OsACOT*, which encodes a thioesterase protein, is a member of Acyl–CoA thioesterase family and can hydrolyze Acyl–CoA into free fatty acids and CoA to regulate lipid metabolism, signal transduction events and gene transcription (Ying *et al*., 2012). At the same time, it has been reported that free fatty acid could make membranes more fluid to exert effects on membrane vesicle transport‐related function, which are the basis for auxin transport (Janda *et al*., 2013). Therefore, free fatty acid could be regarded as potential second messengers in auxin action (Holk and Scherer, 2001). It is believed that, maybe like the function of *OsCOLE1* (CONTINUOUS VASCULAR RING‐LIKE 1), one of the co‐expressed gene of *OsACOT*,* OsACOT* might promote the transport of auxin to enhance the grain filling rate and grain size in OXmACOT transgenic plants (Liu *et al*., 2016). In addition, RNA‐seq data showed the expression level of *OsCOLE1* to be up‐regulated in STTM1432 plants and down‐regulated in OXmiR1432 plants. Very‐long‐chain fatty acid are required for auxin transport and tissue patterning during seed development (Baud *et al*., 2003; Roudier *et al*., 2010). Together with the co‐expressed genes of *OsACOT* during endosperm development and the role of *OsACOT* in fatty acid composition, we speculate that miR1432‐*OsACOT* module may regulate the grain filling rate by mediating auxin transport. Therefore, medium chain fatty acid in regulating seed development may also function as the very‐long‐chain fatty acid.

### Higher contents of IAA and ABA may contribute to miR1432‐OsACOT module mediated rice grain filling improvement

Grain filling is a complex process and regulated by various factors. Plant hormones are crucial regulators of growth and development, especially the processes of cell proliferation and differentiation. Studies have shown that ABA and IAA contributed to rice grain filling through regulating the activities of key enzymes involved in sucrose‐to‐starch conversion in cereal sink organs. Poor grain filling of inferior grains was resulted from low level of ABA and IAA (Zhang *et al*., 2009). ABA influences sugar metabolism and transport through regulation of the expression of some genes encoding components of the sugar response pathway (Gibson, 2004; Rook *et al*., 2006; Zhu *et al*., 2011). Importantly, it is possible that modulating auxin transport could improve grain yield (Kant *et al*., 2009; Liu *et al*., 2015; Qi *et al*., 2008; Takai *et al*., 2013). In present study, we found that expression of genes involved in both ABA signalling, and biosynthesis and auxin and starch metabolism got strengthened in STTM1432 and OXmACOT transgenic plants. The best‐characterized proteins controlling auxin efflux genes, PIN‐FORMED (PINs), were expressed differentially between wild type and miR1432 and its target *OsACOT* transgenic plants, which were consistent with the higher contents of ABA and IAA, suggesting that miR1432 may regulate grain filling through modulating ABA signalling and auxin transport to enhance sucrose to starch metabolism under the altered expression of *OsACOT*.

In summary, miR1432 was found to be a new regulator of grain size determination via controlling rice grain filling through negatively regulating its target, *OsACOT*. Therefore, manipulating the expression of miR1432 or *OsACOT* might be exploited in high‐yield rice breeding. Further investigation is necessary to establish the molecular mechanism of miR1432 and distinct function of *OsACOT* in grain filling.

## Experimental procedures

### Rice cultivation, sampling and dry weights measurement

All experiments were performed using rice (*Oryza sativa* spp. *japonica*) cultivar *Nipponbare*. Wild type and transgenic lines were transplanted in the field under non‐stressed conditions at a research farm of Henan Agricultural University (Henan Province), Songjiang (Shanghai), and Sanya (Hainan Province), during the rice‐growing season. Hulled grain of wild type and transgenic lines were separated from the panicle at 5 DAF (days after fertilization), 10 DAF, 15 DAF, 21 DAF, 27 DAF and 35 DAF. To determine the dry weight, the samples were fast dried at 105 °C for 30 min, and then were kept at 80 °C until constant weight was reached, which indicates a complete dryness of grains.

### Grain trait measurement and grain‐filling rate determination

Phenotypic data were collected at the maturing stage. The hulled grain length, width was measured by an automatic seed‐size‐analysing system (SC‐G, Wanshen, Hangzhou, China) and grain thickness was measured by an electronic digital caliper (SF2000, Guanglu, Guilin, China). Fully filled grains were used for measuring 1,000‐hulled grain weight. The grain filling rates were determined by the followed equations:

(1) $$W=A/{\left(1+{\text{Be}}^{-\;\mathrm{kt}}\right)}^{N}$$

The grain filling rate (*G*) was calculated by the derivative of Equation (1):

(2) $$G=\text{KW}/N[1-{\left(W/A\right)}^{N}]$$

where *W* is the average weight of per grain (mg), *t* is the number of days after fertilization, k, *A*,* B*,* N* are the coefficients determined from regression.

### Plasmid construction and rice transformation

To overexpress miR1432, sequence of pre‐miR1432 from the NCBI (http://www.ncbi.nlm.nih.gov/) with restriction enzyme cutting site *KpnI* and *BamHI* was cloned from DNA of *Nipponbare* and was inserted downstream of the Gt13a promoter (He *et al*., 2011) in pCAMBIA1301. The Short Tandem Target Mimic (STTM) (Tang *et al*., 2012; Yan *et al*., 2012) was used to suppress the expression of OsmiR1432. The STTM fragment with restriction sites *KpnI* and *BamHI* (GGTACCTGTCGGTGTCATTCACTCTCCT GATGTTGTTGTTGTTATGGTCTAATTTAAATATGGTCTAAAGAAGAAGAATTGTCGGTGTCATTCACTCTCCTGATGGATCC) was synthetized by Sangon Biotech (Shanghai, China) and inserted downstream of the Gt13a promoter in pCAMBIA1301. The cDNA of *OsACOT* with seven mismatches to the targeted site by miR1432, without any amino acid changes, was cloned into pCAMBIA1301 and driven by Gt13a promoter. All the constructs were transformed into *Agrobacterium tumefaciens* strain EHA105, and then into rice by Agrobacterium‐mediated transformation (Hiei *et al*., 1997). The full sequence of *OsACOT* was inserted into pHB to confirm the interaction between rice miR1432 and *OsACOT*. The primer sequences of these constructs are listed in Table S6.

### Gene expression analysis

Total RNA was extracted from root, leaf, seedling, panicle, spikelet, and endosperms at 5 DAF, 10 DAF 15 DAF, 21 DAF, 27 DAF and 35 DAF of wild type and at 10 DAF from OXmiR1432, STTM1432 and OXmACOT transgenic plants with Trizol reagent (Invitrogen) following the manufacturer's instructions. Stem‐loop qPCR was used to detect and quantify the mature miR1432 during growth stages and grain filling. In brief, miRNAs were reverse transcribed into cDNAs using a miRNA specific stem‐loop reverse transcription primer and a reverse transcriptase enzyme (Promega, Madison, WI). Quantitative real‐time polymerase chain reaction (qRT‐PCR) was performed to analyse transcript abundance of *OsACOT* and other genes. In the qPCR, a 5 μL aliquot of 1:20 diluted cDNA was used as the template in a 20 μL PCR reaction system using SYBR green reaction mix (SYBR Green QRT‐PCR Master Mix; Toyobo). The reaction process included a pre‐incubation at 95 °C for 5 min, followed by 40 cycles of denaturation at 95 °C for15 s, annealing at 60 °C for 15 s, and extension at 72 °C for 32 s, in a BioRad iQ5 sequence detection system (BioRad, Hercules, CA). Rice β‐actin gene was used as the internal control, and all PCR reactions were repeated three times.

### Northern blot hybridization analysis

Approximately 30 μg of total RNA was separated on 18% polyacrylamide denaturing gels, using rice miR1432 RNA oligonucleotide as marker. RNAs were transferred to Amersham HybondTM‐N+ membrane (GE Healthcare, Amersham, UK) and hybridized with a locked nucleic acid DNA oligonucleotide complementary to the miR1432 sequence, which had been labelled with γ‐32pATP. Blots were prehybridized and hybridized at 42 °C in 125 mm NaHPO4 (pH 7.2), 250 mm NaCl_2_, 7% SDS and 50% formamide, and washed at 42 °C twice with 2 × SSC, 0.2% SDS followed by a higher stringency wash of 1 × SSC, 0.1% SDS at 37 °C. Blots were imaged using an FLA‐5000 phosphorimager (Fuji Medical Systems Inc., Stamford, CT). U6 was used as a loading control. Sequence of probe is listed in Table S6.

### GUS staining

Vector pCAMBIA1301 was used to construct the GUS‐miR1432 transgenic plants. About 2500 bp region upstream of the miR1432 was used as the promoter region to drive the expression of GUS gene. The GUS activity in the transgenic plants was localized by histochemical staining with 5‐bromo‐4‐chloro‐3‐indoly‐β‐glucuronic acid (X‐Gluc). Tissues at different stages were collected and incubated for about 3 h at 37 °C in staining buffer. Primers used to clone the promoter of miR1432 are listed in Table S6.

### Prediction of miR1432 targets

The potential miRNA targets were predicted using the psRNATarget program (http://bioinfo3.noble.org/psRNATarget/) with default parameters. The sequence of miR1432 was used as a custom sequence. The *O. sativa* TIGR genome cDNA OSA1 Release 5 (OSA1R5) was used as the genomic library for the search target.

### Verification of interaction between miR1432 and its target

Agroinfilitration of *Nicotiana benthamiana* leaves as a transient expression method (English, 1997) was used to validate the interaction between miRNA and its putative targets *in vivo*. In brief, predictive targets and precursor of miR1432 were constructed in the same vector respectively. And non‐sequence was used as control. Then *A. tumefaciens,* with the 35S promoter driving the expression of precursor of miR1432 and its target, was infiltrated into the leaves of *Nicotiana benthamiana*. If the interaction between miR1432 and its putative targets existed, the transcripts level of target would decrease when compared with putative targets fused with empty vector.

### 5′ RNA ligase‐mediated rapid amplification (RACE)

Total RNA of wild‐type endosperm at 10 DAF was directly ligated to an RNA adaptor (CCTCTAGAGATTCGCGGATCCACAGCC TACTGATGATCAGTCGATGG), which has a 5′‐ hydroxyl group at both ends and, thus, can only ligate to 5′ ‐phosphorylated RNA, including the truncated products of miRNA‐guided mRNA cleavage. The ligated product was directly reverse‐transcribed with an oligo (dT) primer. The cDNA was amplified by nested PCR, and the fiAF was directly ligated to an RNA adaptor (CCTCTA GAGATTCGCGGATCCACAGCCTACTGATGATCAGTCGATGG), which has a 5′‐ hydroxyl group at both *OsACOT* (*LOC_Os04g35590*) are shown in Table S6.

### Analysis of Gene Ontology of different expressed genes

Endosperms of wild type, STTM1432 and OXmiR1432 at 10 DAF were collected for total RNA extraction. Library construction was conducted with a NEB Next Multiplex Library Prep Set for Illumina (NEB) and then sequenced on an Illumina Hiseq2000 Platform. Number of fragments per kilobase exon per million fragments (FPKM) was calculated before gene expression analysis. Genes which were down‐regulated in OXmiR1432 seeds and up‐regulated in STTM1432 seeds, compared to WT, by at least 1.5‐fold were subjected to further GO enrichment analysis. BiNGO was used to analyse item classification of differentially expressed genes (Maere *et al*., 2005). *P*‐value of each GO item classification was calculated by hypergeometric test and was further checked by means of Benjamin & Hochberg. GO item classification involved at least five genes and checked *P*‐value, < 0.05, was considered enriched, significantly.

### Measurements of fatty acid

Identification and quantification of fatty acid in brown rice was conducted by GC‐MS by assaying fatty acid methyl esters (FAMEs). Mature grain of wild type and OXmACOT transgenic plants were collected and grounded into power. 50 mg of each sample was weighted to determine the fatty acid content. Thirty‐seven kinds of FAMEs were prepared as previously described with a few modifications. First, 1 mL chloroform was added into the power and ultrasounded for 30 min, the methyl esterified with 1% sulphuric acid‐methanol for half an hour. 25 μL internal quantitative standard with 1 mL N‐hexane was added in every sample. Subsequently, the shaken sample was prepared for GC‐MS analysis. The Agilent 7890A/5975c CG/MS system (Agilent, Santa Clara, CA) was used in the GC‐MS analysis. The concentrations of individual fatty acid were expressed as percentages of the sum of all identified fatty acid contents. Three biology repeats of grain samples were analysed.

### Phytohormone measurements

Contents of auxin (IAA) and abscisic acid (ABA) were quantified by high‐performance liquid chromatography‐tandem mass spectrometry (ESI‐HPLC‐MS/MS) as previously reported (Xu *et al*., 2016). Briefly, for accuracy quantitative results, 0.3 g sample from endosperms was collected and grounded into powder using liquid nitrogen. Then 3 mL isopropanol/hydrochloric acid extraction buffer was added into the powder, followed by shaking at 4 °C for 30 min. After that 5 mL dichloromethane was added and the samples were shaken at 4 °C for 30 min. IAA and ABA were dissolved in the lower organic phase after centrifuging at 4 °C for 5 min. Organic phase was dried by nitrogen protected from light and then dissolved by 400 μL methanol with 0.1% formic acid. HPLC‐MS/MS analysis was conducted after filtering with 0.2 μm filter membrane. HPLC‐MS/MS system consists of an Aglient1290 HPLC system (Agilent), a SCIEX‐6500Qtrap (MSMS, AB) with an ESI source.

## Conflict of interest

The authors declare no conflict of interests.

## Supporting information


**Figure S1** Study of transgenic rice with altered expression of miR1432 (in the year 2016‐Shanghai).
**Figure S2** Morphologies of *Nipponbare* (WT), STTM1432 and OXmiR1432 transgenic plants.
**Figure S3** Study of transgenic plant expressing *OsmACOT* (in the year 2017‐Hainan).
**Figure S4** Morphologies of *Nipponbare* (WT) and OXmACOT transgenic plants.
**Figure S5** Correlation analysis of expression level of *OsACOT* and grain weight.
**Figure S6** Enrichment analysis of *OsACOT* co‐expressed genes.
**Figure S7** Validation of *OsACOT* co‐expressed genes.
**Figure S8** GO enrichment analysis of differentially expressed genes (DEGs) in endosperm of *Nipponbare* (WT) and miR1432 transgenic plants.
**Figure S9** Orthologs of *OsACOT* in different species.

**Table S1** Grain yield and associated components of main panicle in miR1432 transgenic plants in field trials in the year 2015 (Zhengzhou) and 2016 (Shanghai).
**Table S2** Grain filling parameters for wild‐type and different transgenic plants of miR1432 in year the 2015 (Zhengzhou) and 2016 (Shanghai).
**Table S3** Grain filling parameters for wild‐type and different transgenic of *OsACOT* in the year 2017 (Hainan).
**Table S4** Potential targets of rice miR1432 predicted by psRNATarget.
**Table S5** Expression analysis of potential targets of rice miR1432 in STTM1432 and OXmiR1432 transgenic plants by RNA‐seq.
**Table S6** Primers used in the study.
