# Supplementary material for: miR1432‐OsACOT (Acyl‐CoA thioesterase) module determines grain yield via enhancing grain filling rate in rice
Source: Plant Biotechnol J. 2018 Oct 8;17(4):712–23. doi: 10.1111/pbi.13009 (PMC6419572; doi:10.1111/pbi.13009)
Supplement: Supplementary file 1 — Figure S1 Study of transgenic rice with altered expression of miR1432 (in the year 2016‐Shanghai). Figure S2 Morphologies of Nipponbare (WT), STTM1432 and OXmiR1432 transgenic plants. Figure S3 Study of transgenic plant expressing OsmACOT (in the year 2017‐Hainan). Figure S4 Morphologies of Nipponbare (WT) and OXmACOT transgenic plants. Figure S5 Correlation analysis of expression level of OsACOT and grain weight. Figure S6 Enrichment analysis of OsACOT co‐expressed genes. Figure S7 Validation of OsACOT co‐expressed genes. Figure S8 GO enrichment analysis of differentially expressed genes (DEGs) in endosperm of Nipponbare (WT) and miR1432 transgenic plants. Figure S9 Orthologs of OsACOT in different species. [file PBI-17-712-s001.docx]

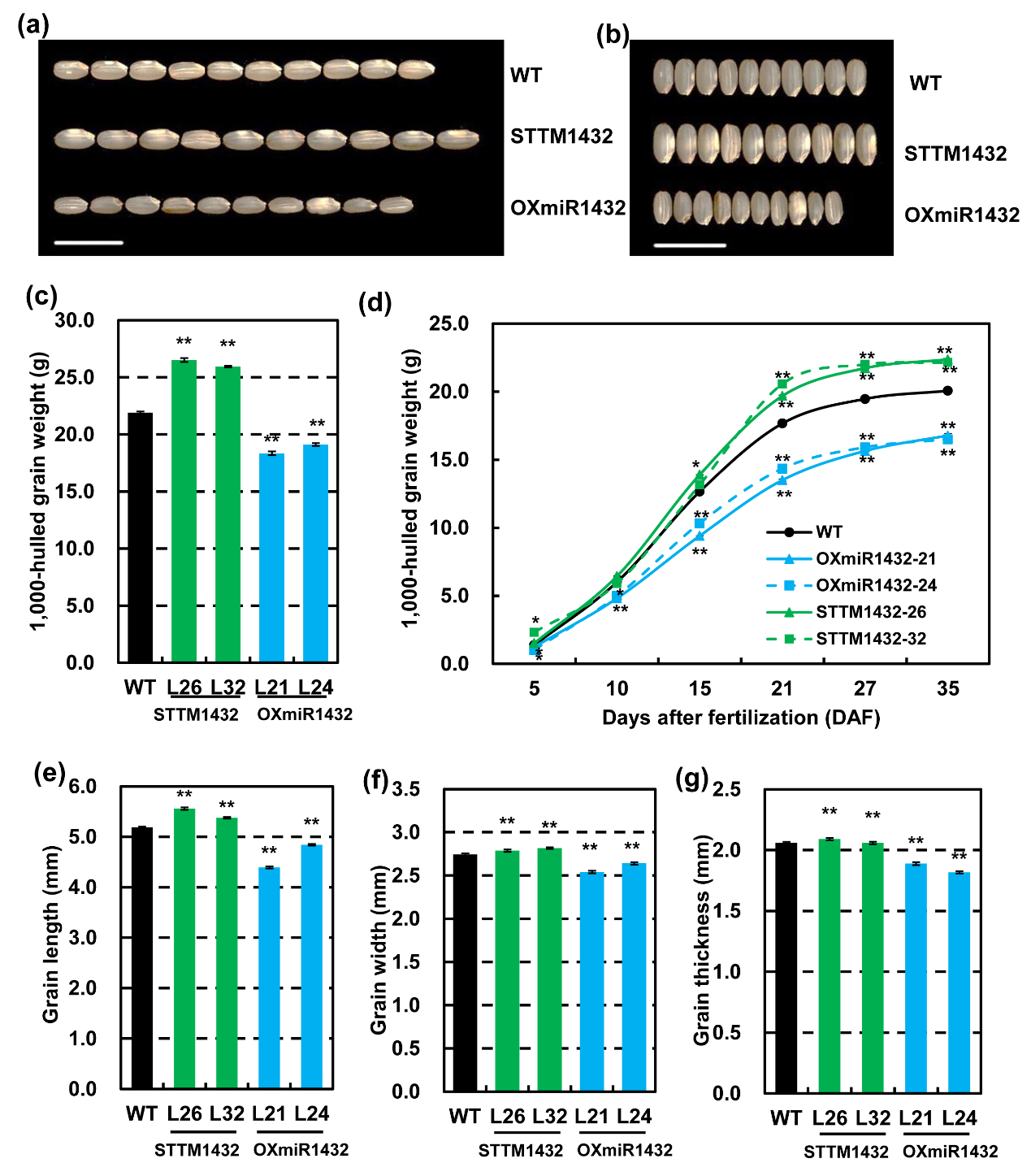


**Figure S1****.** Study of transgenic rice with altered expression of miR1432 (in the year 2016-Shanghai). (a-b) Phenotypic observation of grain size of *Nipponbare* (WT), STTM1432 and OXmiR1432 transgenic plants. Scale bars, 5 mm; (c) Measurement of the 1,000-hulled grain weight of *Nipponbare* (WT), STTM1432, and OXmiR1432 transgenic plants; (d) Measurements of grain weight of *Nipponbare* (WT), STTM1432 and OXmiR1432 transgenic plants during grain filling; (e**-**g) Detailed analysis of grain traits including grain length (e), width (f), and thickness (g) of *Nipponbare* (WT) and miR1432 transgenic plants; Experiments were repeated three times and data are presented as mean ± SD (*n*=1000 grains). Statistical analysis was performed by Student’s t-test (**, *P*<0.01; *, *P*<0.05).


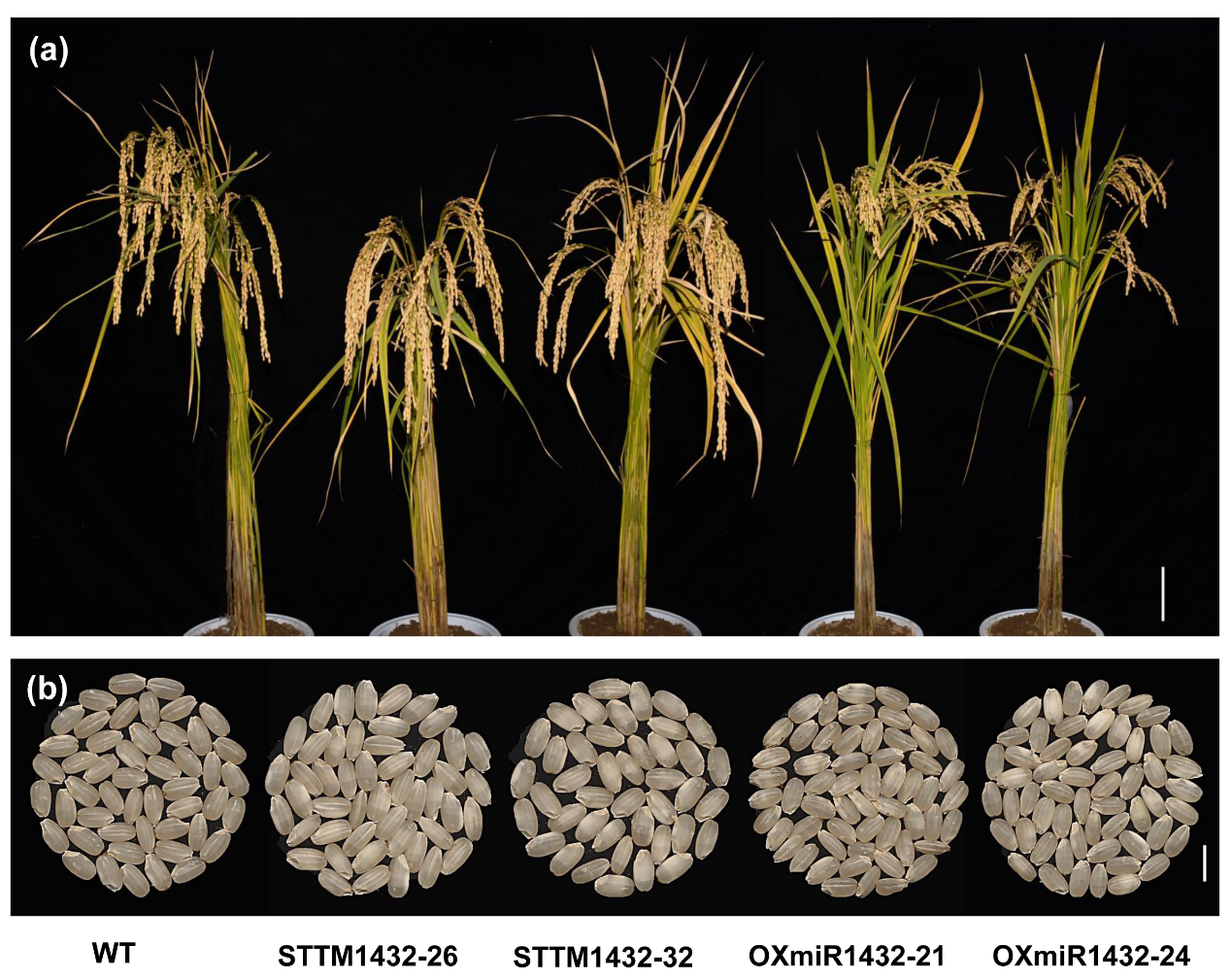


**Figure S2.** Morphologies of *Nipponbare* (WT), STTM1432 and OXmiR1432 transgenic plants. (a) Photos of wild type plants and miR1432 transgenic plants at harvest stage in the field; scale bar, 10cm; (b) Hulled grains of wild type and miR1432 transgenic plants; scale bar, 0.5cm.


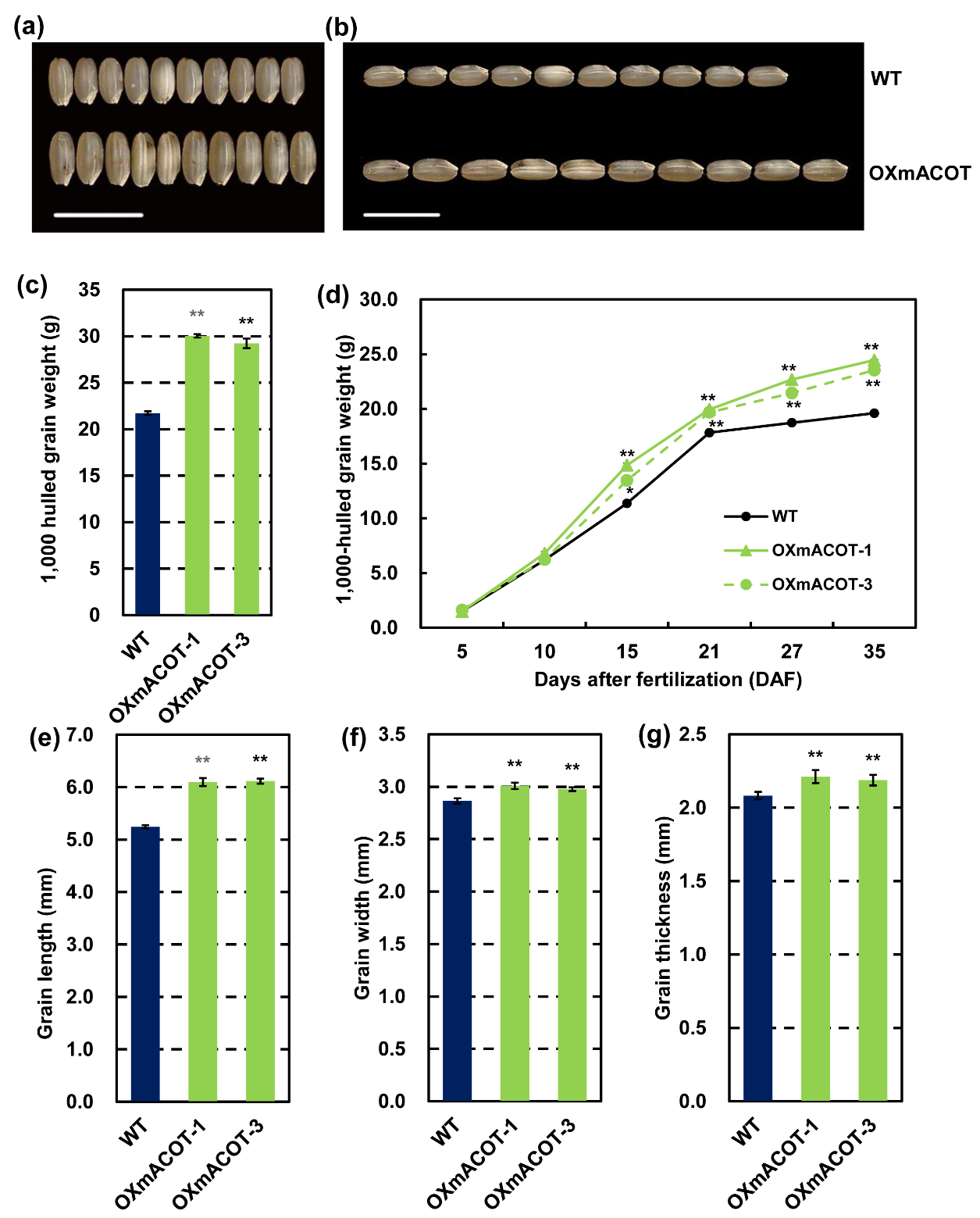


**Figure S3.** Study of transgenic plant expressing *OsmACOT* (in the year 2017-Hainan). (a-b) Phenotypic observation of *Nipponbare* (WT) and OXmACOT grains. Scale bars, 1 cm; (c) Expressions levels of *OsACOT* in endosperm at 10 DAF (days after fertilization) of *Nipponbare* (WT) and OXmACOT plants; (d) Measurements of grain weight of *Nipponbare* (WT) and OXmACOT transgenic plants during grain filling; (e-g) Detailed analysis of grain traits including grain length (f), width (g) and thickness (h). Experiments were repeated three times and data are presented as mean ± SD (*n*=1000 grains); statistical analysis was performed by Student’s t-test (**, *P*<0.01; *, *P*<0.05).


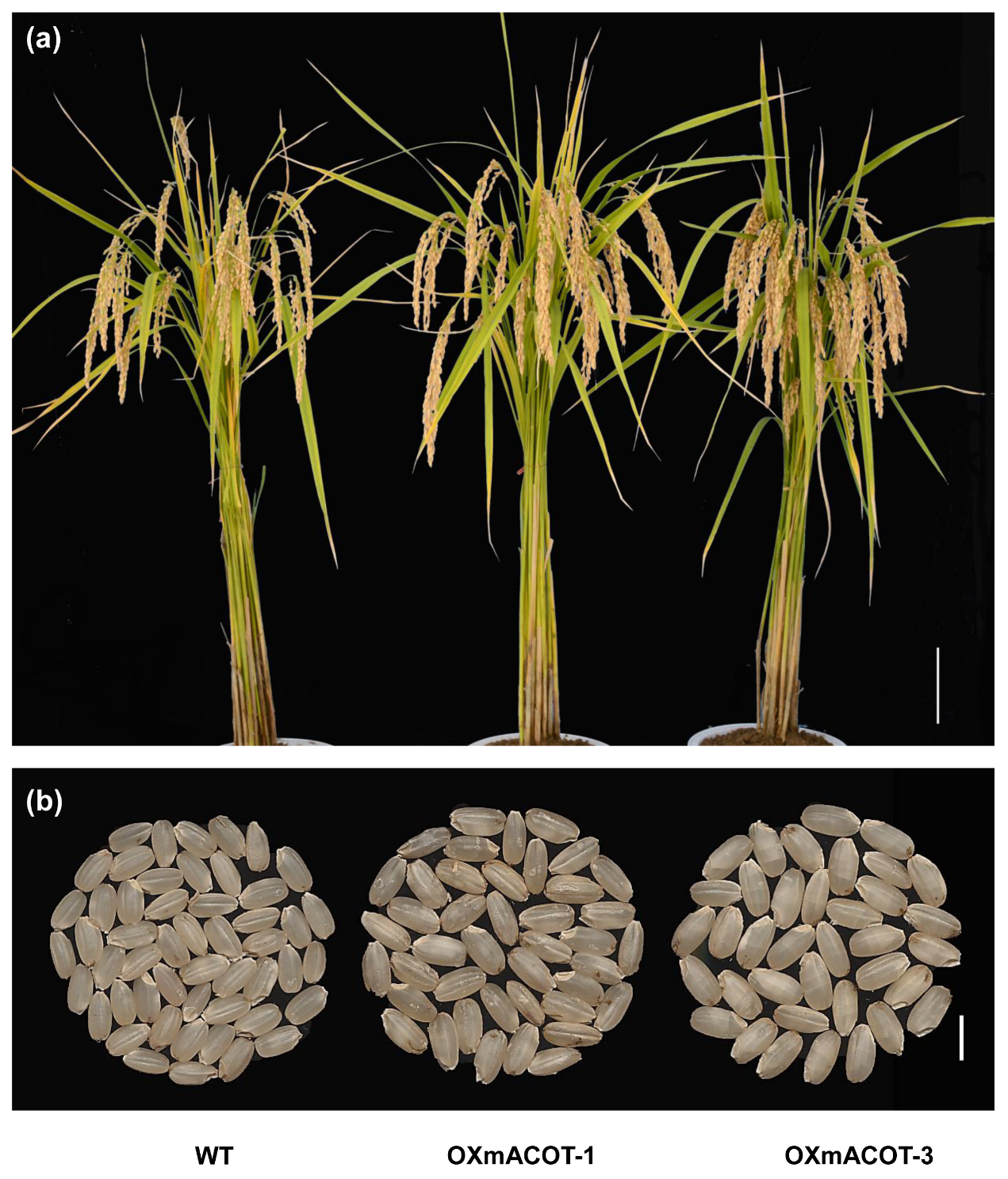


**Figure S4.** Morphologies of *Nipponbare* (WT) and OXmACOT transgenic plants. (a) Photos of wild type plants and OXmACOT transgenic plants at harvest stage in the field; scale bar, 10cm; (b) Hulled grains of wild type plants and OXmACOT transgenic plants; scale bar, 0.5cm.


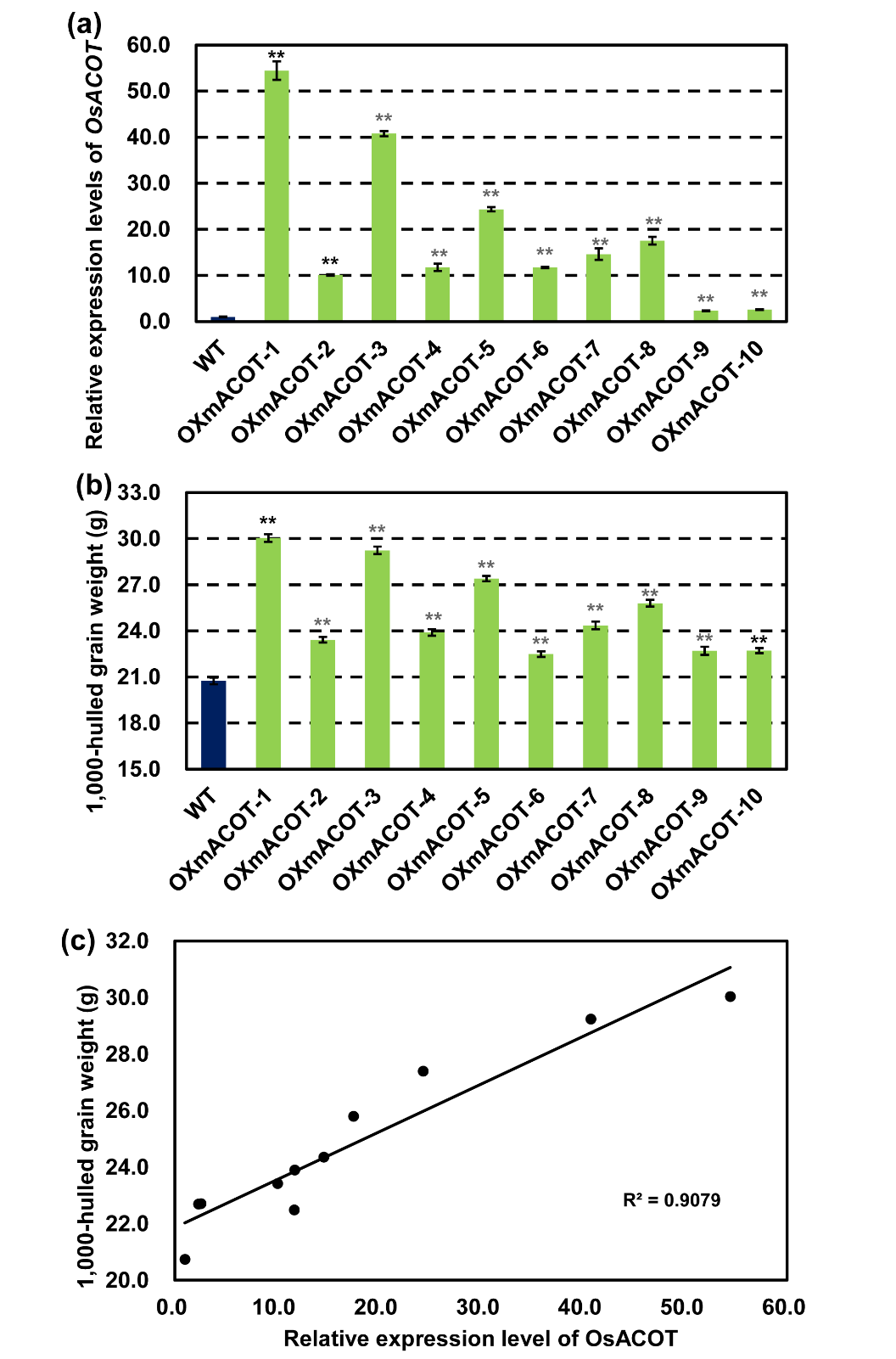


**Figure S5.** Correlation analysis of expression level of *OsACOT* and grain weight. (a) The expression levels of *OsACOT* in wild type and different OXmACOT transgenic lines; (b) 1,000-hulled grain weight of wild type and different OXmACOT transgenic lines; (c) The positive correlation of the dosage of *OsACOT* mRNA and the grain weight in OXmACOT transgenic plants from the ten lines (OXmACOT line 1- 10). Rice endosperms at 10 DAF (days after fertilization) were used for analysis; experiments were repeated three times and data are shown as means± SD; statistical analysis was performed by Student’s t-test (**, *P*<0.01; *, *P*<0.05).


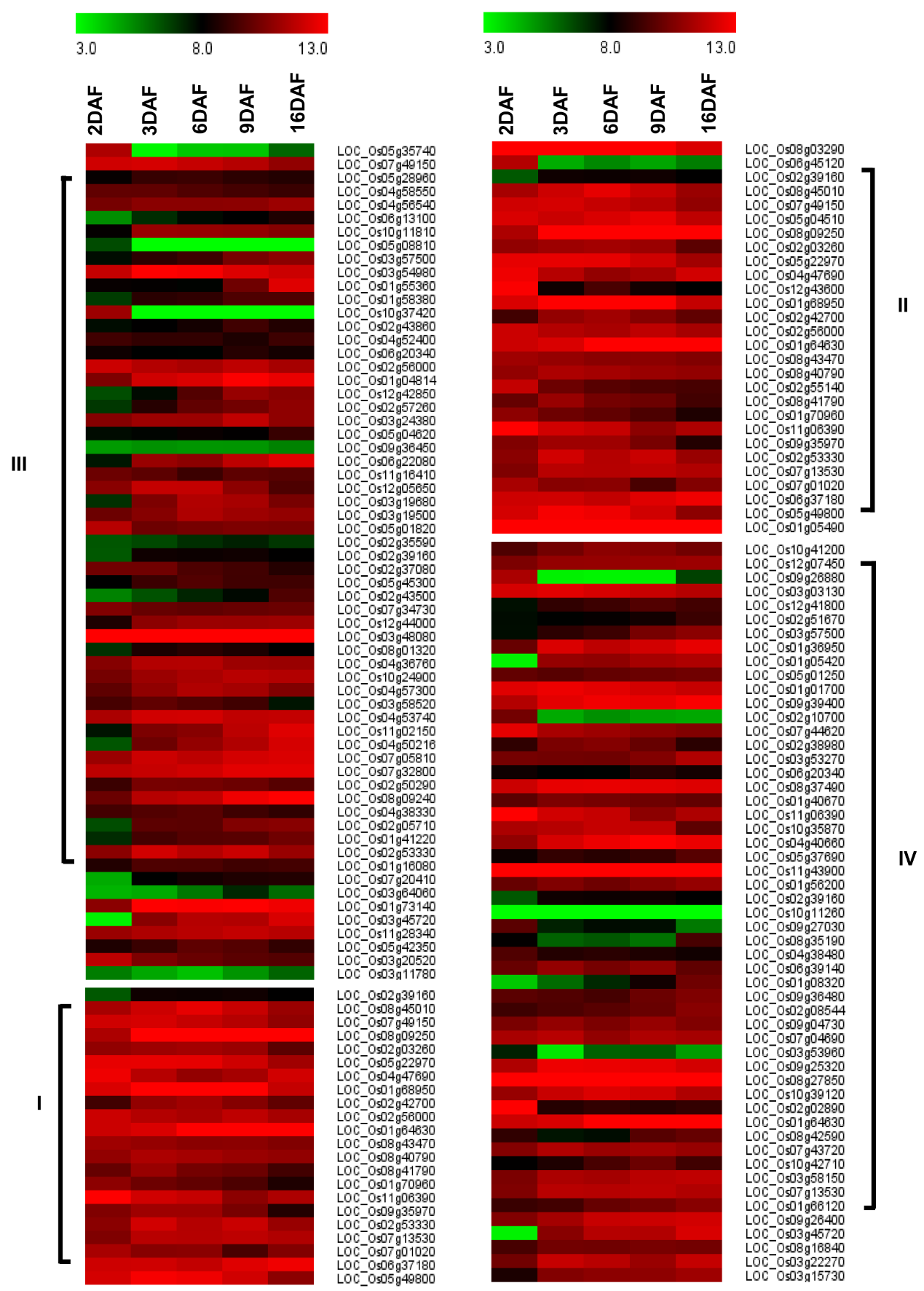


**Figure S6.** Enrichment analysis of *OsACOT* co-expressed genes. Expressions of genes during rice endosperm development were analyzed from microarray data and genes involved in (I) glucose metabolism; (II) endomembrane organization; (III) Lipid biosynthesis, (IV) response to hormones and ATP synthesis were analyzed; DAF represents days after fertilization.


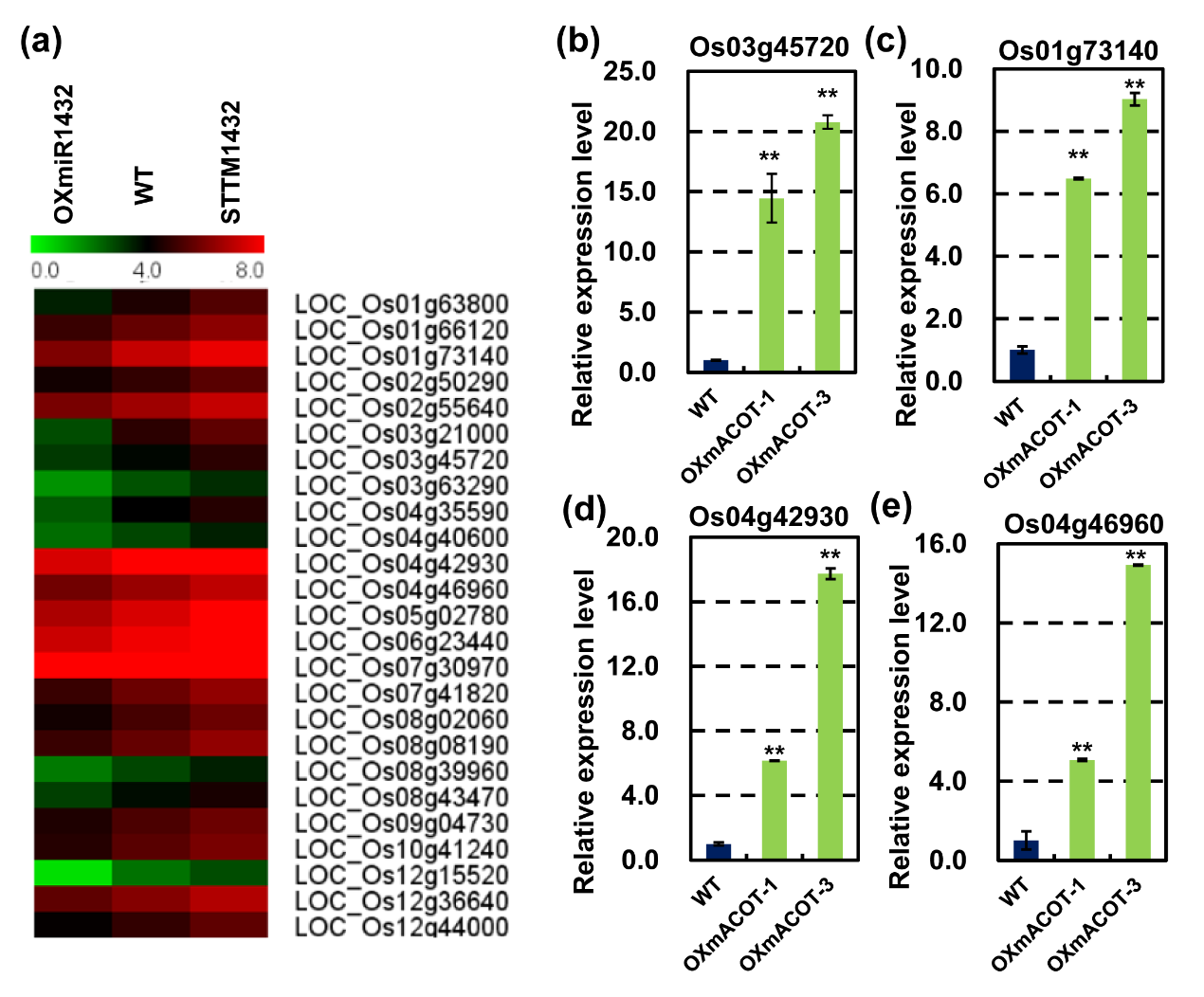


**Figure S7.** Validation of *OsACOT* co-expressed genes. (a) Expression levels of *OsACOT* co-expressed genes in OXmiR1432 and STTM1432 transgenic plants detected by RNA-seq analysis; (b-e) Validation of some co-expressed genes in OXmACOT plants by qPCR. Rice endosperms at 10 DAF (days after fertilization) were used for analysis; experiments were repeated three times and data are shown as means± SD; statistical analysis was performed by Student’s t-test (**, *P*<0.01; *, *P*<0.05).


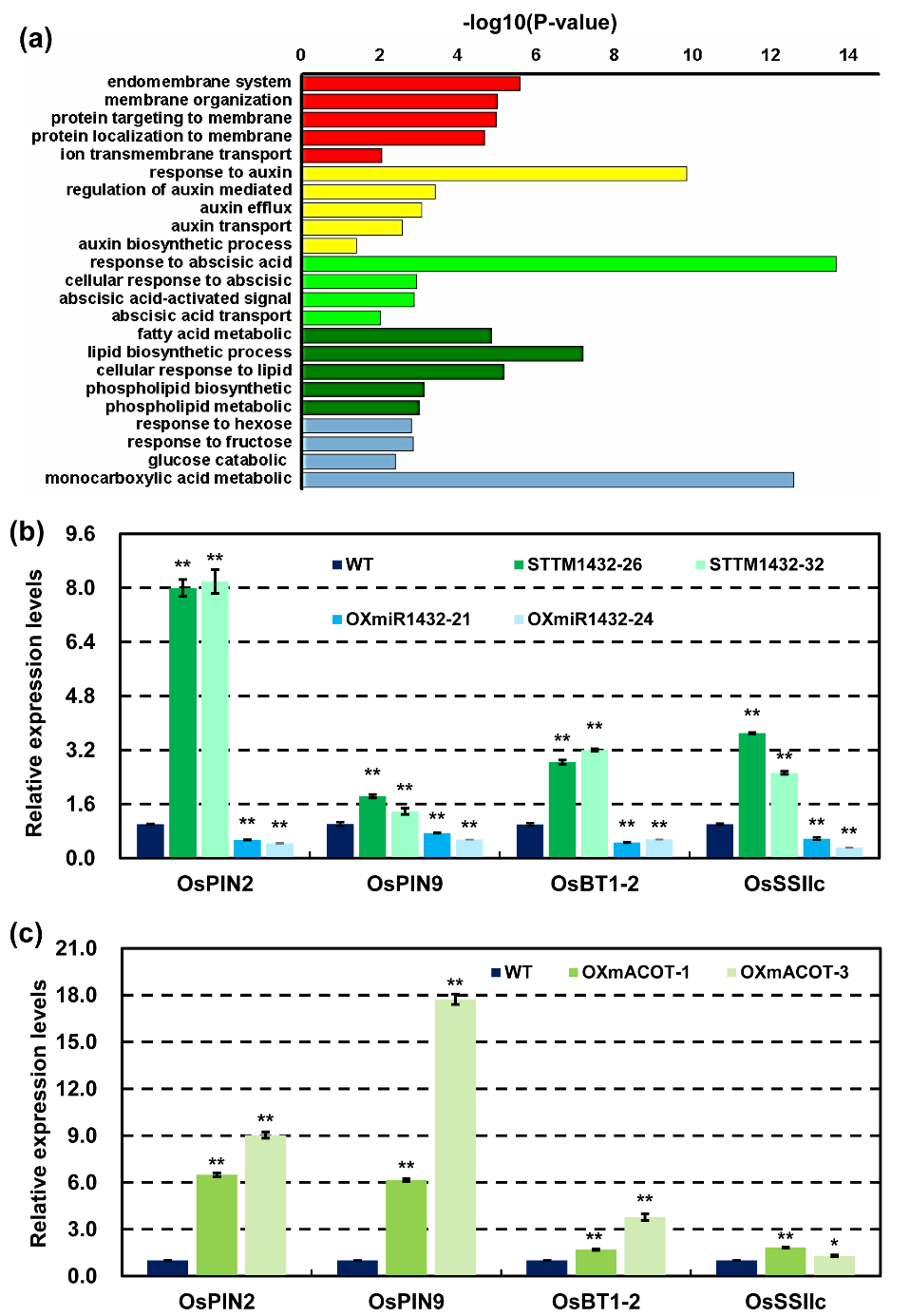


**Figure S8.** GO enrichment analysis of differentially expressed genes (DEGs) in endosperm of *Nipponbare* (WT) and miR1432 transgenic plants. (a) GO items analysis of DEGs. Genes down-regulated in OXmiR1432 seeds and up-regulated in STTM1432 seeds by at least 1.5-fold, compared to *Nipponbare* (WT), were identified and subjected to further analysis. Only GO terms with a corrected *P*-value <0.05 and at least 5 annotated genes were retained. Length of bars represents negative logarithm (base 10) of the corrected *P*-value; (b-c) qRT-PCR expression analysis of genes involved in starch synthesis and auxin transport in STTM1432 and OXmiR1432 (b), and OXmACOT (c) transgenic lines. Rice endosperms at 10 DAF (days after fertilization) were used for analysis; experiments were repeated three times and data are shown as means± SD; statistical analysis was performed by Student’s t-test (**, *P*<0.01; *, *P*<0.05).


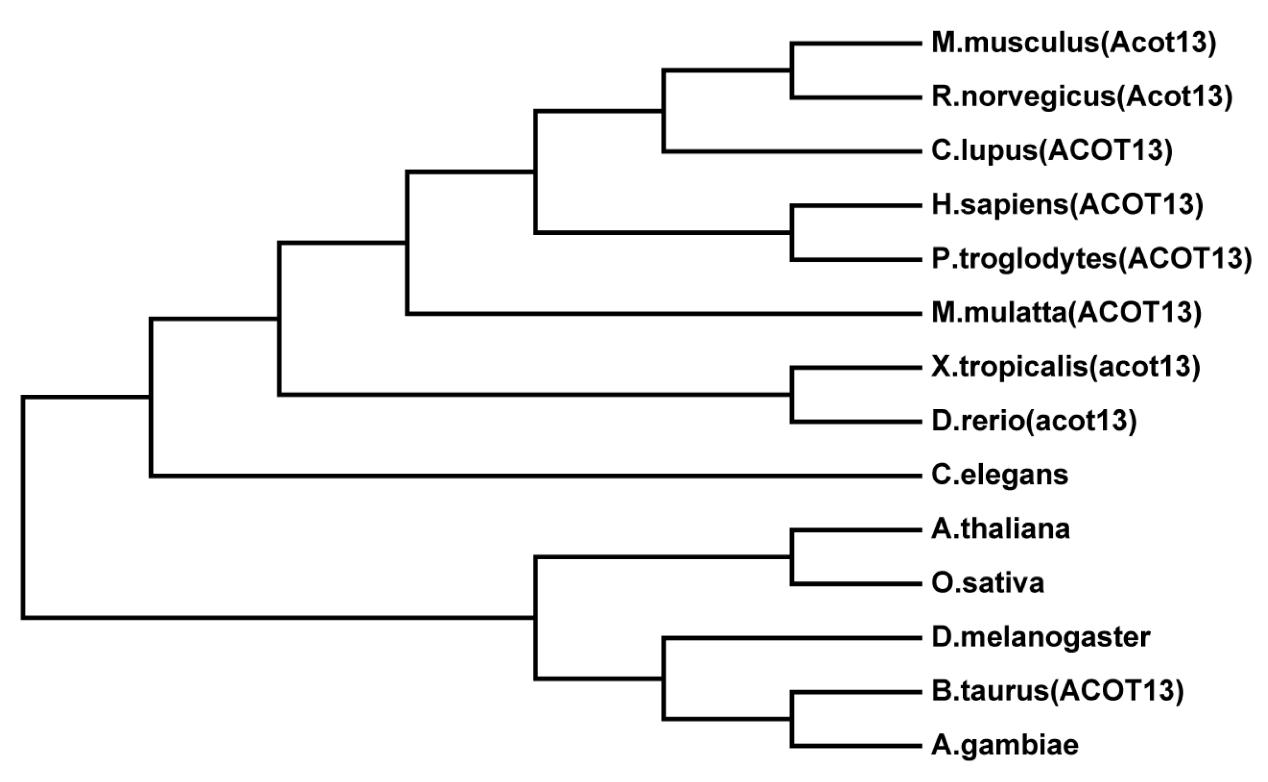


**Figure S9.** Orthologs of *OsACOT* in different species. Phylogenetic analysis of *OsACOT* orthologs was conducted using MEGA 5. The amino acid sequences of 13 *OsACOT* orthologs were used to establish a bootstrap M-L phylogenetic tree. 1,000 replicates were performed to determine the statistical support for each node.
