# Supplementary material for: miR1432‐OsACOT (Acyl‐CoA thioesterase) module determines grain yield via enhancing grain filling rate in rice
Source: Plant Biotechnol J. 2018 Oct 8;17(4):712–23. doi: 10.1111/pbi.13009 (PMC6419572; doi:10.1111/pbi.13009)
Supplement: Supplementary file 2 — Table S1 Grain yield and associated components of main panicle in miR1432 transgenic plants in field trials in the year 2015 (Zhengzhou) and 2016 (Shanghai). Table S2 Grain filling parameters for wild‐type and different transgenic plants of miR1432 in year the 2015 (Zhengzhou) and 2016 (Shanghai). Table S3 Grain filling parameters for wild‐type and different transgenic of OsACOT in the year 2017 (Hainan). Table S4 Potential targets of rice miR1432 predicted by psRNATarget. Table S5 Expression analysis of potential targets of rice miR1432 in STTM1432 and OXmiR1432 transgenic plants by RNA‐seq. Table S6 Primers used in the study. [file PBI-17-712-s002.docx]

**Table S1.** Grain yield and associated components of main panicle in miR1432 transgenic plants in field trials.

| Year/Sites | Characters | WT | OXmiR1432 | |  | STTM1432 | |
| --- | --- | --- | --- | --- | --- | --- | --- |
|  |  |  | L21 | L24 |  | L26 | L32 |
| 2015 | Length of main panicle (cm) | 21.21±0.12 | 20.46±0.13 | 20.87±0.32 |  | 21.15±0.30 | 20.78±0.36 |
| Zhengzhou | Tiller number | 14.02±0.59 | 13.76±0.32 | 13.65±0.47 |  | 13.71±0.18 | 13.88±0.34 |
|  | Primary branch numbers | 11.45±0.30 | 11.38±0.21 | 11.51±0.09 |  | 11.64±0.17 | 11.68±0.35 |
|  | Secondary branch numbers | 21.14±1.60 | 20.85±0.34 | 20.56±0.19 |  | 20.72±1.43 | 20.34±1.21 |
|  | Spikelet number | 130.26±3.45 | 121.96±1.92 | 125.63±6.20 |  | 125.91±0.48 | 129.31±7.00 |
|  | Seed-setting rate (%) | 91.09±3.02 | 90.56±4.10 | 90.89±3.04 |  | 91.93±1.86 | 91.65±1.30 |
|  | 1,000 hulled grain weight (g) | 21.92±0.14 | 18.31±0.22** | 19.15±0.17** |  | 26.53±0.24** | 25.94±0.19** |
|  | Grain yield per plant (g) | 36.50±2.21 | 27.12±1.07** | 28.50±1.06** |  | 43.30±1.14** | 42.52±2.36** |
|  | Yield increase (%) | —— | -25.70 | -21.92 |  | 18.63 | 16.49 |
| 2016 | Length of main panicle (cm) | 18.44±0.21 | 18.26±0.23 | 18.64±0.26 |  | 19.10±0.21 | 18.53±0.26 |
| Shanghai | Tiller number | 14.31±0.20 | 13.18±0.72 | 14.23±0.25 |  | 14.32±0.34 | 13.74±0.19 |
|  | Primary branch numbers | 8.95±0.23 | 8.27±0.21 | 9.01±0.36 |  | 9.20±0.15 | 8.96±0.18 |
|  | Secondary branch numbers | 15.38±0.64 | 14.80±0.51 | 14.83±0.62 |  | 14.67±0.63 | 13.59±0.42 |
|  | Spikelet number | 96.52±3.60 | 91.29±2.65 | 102.50±2.55 |  | 100.14±2.42 | 99.80±1.83 |
|  | Seed-setting rate (%) | 89.82±0.91 | 90.71±1.14 | 87.28±1.25 |  | 89.76±1.14 | 89.79±0.73 |
|  | 1,000 hulled grain weight (g) | 20.30±0.14 | 14.12±0.73** | 14.59±0.67** |  | 24.55±0.64** | 24.84±0.49** |
|  | Grain yield per plant (g) | 24.20±1.15 | 15.42±0.56** | 18.37±0.46** |  | 31.50±0.42** | 30.25±1.71** |
|  | Yield increase (%) | — | -36.58 | -24.09 |  | 30.16 | 25.00 |

Values shown are the mean ± SD (*n*=10 plants, *n*=60). Significant differences were identified using Student’s t-test. ^*^ *P*<0.05, ^**^*P*<0.01.

**Table S2.** Grain filling parameters for wild-type and different transgenic plants of miR1432 in year 2015 (Zhengzhou) and year 2016 (Shanghai).

| Year/Sites | Parameters | WT | OXmiR1432 | |  |  | STTM1432 | |
| --- | --- | --- | --- | --- | --- | --- | --- | --- |
|  |  |  | L21 | L24 |  |  | L26 | L32 |
| 2015 | GR0 | 1.33±0.21 | 2.11±0.60 | 1.17±0.24 |  |  | 1.25±0.17 | 0.98±0.35 |
| Zhengzhou | Vmax (mg/(grain·d)) | 1.30±0.01 | 0.90±0.05** | 1.08±0.05** |  |  | 1.41±0.06* | 1.70±0.06* |
|  | Va (mg/(grain·d)) | 0.93±0.03 | 0.64±0.02** | 0.76±0.07* |  |  | 1.05±0.03* | 1.10±0.06* |
|  | tmax (d) | 11.63±0.72 | 11.71±0.35 | 11.60±0.70 |  |  | 12.39±0.75 | 14.12±0.24 |
|  | Active period (d) | 21.61±0.71 | 24.54±1.77 | 22.17±1.12 |  |  | 21.51±0.74 | 22.24±0.59 |
| 2016 | GR0 | 7.92±0.49 | 3.37±0.14** | 1.81±0.15** |  |  | 5.94±0.95* | 7.52±0.15 |
| Shanghai | Vmax (mg/(grain·d)) | 1.36±0.01 | 0.88±0.01** | 0.89±0.01** |  |  | 1.66±0.01** | 1.58±0.01** |
|  | Va (mg/(grain·d)) | 0.91±0.01 | 0.60±0.00** | 0.62±0.01** |  |  | 1.16±0.01** | 1.09±0.01** |
|  | tmax (d) | 8.28±0.06 | 10.09±0.12** | 9.96±0.09** |  |  | 8.65±0.05 | 8.98±0.14 |
|  | Active period (d) | 20.54±0.14 | 23.86±0.11* | 24.09±0.28* |  |  | 21.71±0.16 | 22.83±0.22 |

GR0: Initial filling potential; Vmax: Maximum filling rate; Va: Mean filling rate; tmax: Date when reaches maximum filling rate; Active period: Days of active grain filling. Values shown are the mean ± SD (*n*=60). Significant differences were identified using Student’s t-test. ^*^ *P*<0.05, ^**^*P*<0.01.

**Table S3.** Grain filling parameters for wild-type and different transgenic of *OsACOT* in the year 2017 (Hainan).

| Year/Sites | Parameters | WT | OXmACOT-1 | OXmACOT-3 |
| --- | --- | --- | --- | --- |
| 2017 | GR0 | 0.99±0.18 | 1.89±0.12* | 1.14±0.10* |
| Hainan | Vmax (mg/(grain·d)) | 1.25±0.03 | 1.46±0.01** | 1.54±0.03** |
|  | Va (mg/(grain·d)) | 0.87±0.02 | 1.03±0.01** | 1.09±0.04* |
|  | tmax (d) | 13.30±0.46 | 11.60±0.13 | 12.46±0.46 |
|  | Active period (d) | 22.51±0.66 | 23.71±0.33 | 23.92±1.12 |

GR0: Initial filling potential; Vmax: Maximum filling rate; Va: Mean filling rate; tmax: Date when reaches maximum filling rate; Active period: Days of active grain filling. Values shown are the mean ± SD (*n*=60). Significant differences were identified using Student’s t-test. ^*^ *P*<0.05, ^**^*P*<0.01.

**Table S4.** Potential targets of rice miR1432 predicted by psRNATarget.

| **Target ID** | **Expectation** | **UPE** | **Inhibition** | **Description** |
| --- | --- | --- | --- | --- |
| LOC_Os03g59790 | 0.5 | 23.50 | Cleavage | calcium-binding protein |
| LOC_Os03g59770 | 0.5 | 20.60 | Cleavage | calcium-binding allergen Ole e 8 |
| LOC_Os05g07030 | 2.5 | 15.98 | Cleavage | arginyl-tRNA synthetase |
| LOC_Os03g16110 | 2.5 | 19.18 | Cleavage | serine/threonine-protein phosphatase PP1 |
| LOC_Os05g06360 | 2.5 | 17.14 | Translation | expressed protein |
| **LOC_Os04g35590(*OsACOT)*** | **3.0** | **19.58** | **Cleavage** | **thioesterase superfamily member 2** |
| LOC_Os05g07210 | 3.0 | 11.00 | Cleavage | ZIP zinc/iron transport family protein |
| LOC_Os01g07600 | 3.0 | 20.03 | Cleavage | expressed protein |
| LOC_Os08g36910 | 3.0 | 11.97 | Cleavage | alpha-amylase isozyme 3D precursor |
| LOC_Os07g47340 | 3.0 | 17.96 | Cleavage | expressed protein |
| LOC_Os08g20410 | 3.0 | 22.22 | Translation | expressed protein |
| LOC_Os05g41480 | 3.0 | 23.55 | Cleavage | plastidic phosphate translocator-like protein1 |
| LOC_Os07g12240 | 3.0 | 16.62 | Translation | calmodulin TaCaM2-1, putative |
| LOC_Os06g13820 | 3.0 | 21.23 | Translation | dynamin-2A, putative |
| LOC_Os01g51754 | 3.0 | 21.67 | Translation | alpha-amylase isozyme C2 precursor |
| LOC_Os07g39780 | 3.0 | 15.35 | Translation | ubiquitin-like 1-activating enzyme E1B |

**Table S5.** Expression analysis of potential targets of rice miR1432 in STTM1432 and OXmiR1432 transgenic plants by RNA-seq.

| **Target ID** | **Expressions (FPKM)** | | |
| --- | --- | --- | --- |
|  | **OXmiR1432** | **WT** | **STTM1432** |
| LOC_Os03g59790 | None | None | None |
| LOC_Os03g59770 | 0 | 0.2 | 0.19 |
| LOC_Os05g07030 | 36.71 | 45.45 | 34.94 |
| LOC_Os03g16110 | 79.04 | 129.74 | 105.27 |
| LOC_Os05g06360 | 5.51 | 7.04 | 5.59 |
| **LOC_Os04g35590(*OsACOT)*** | **5.87** | **16.42** | **24.35** |
| LOC_Os05g07210 | 1.57 | 5.89 | 4.18 |
| LOC_Os01g07600 | 0.72 | 1.38 | 0.92 |
| LOC_Os08g36910 | None | None | None |
| LOC_Os07g47340 | 27.74 | 27.79 | 44.04 |
| LOC_Os08g20410 | 0.14 | 0.14 | 0.42 |
| LOC_Os05g41480 | 28.43 | 42.15 | 46.11 |
| LOC_Os07g12240 | None | None | None |
| LOC_Os06g13820 | 39.74 | 38.43 | 24.55 |
| LOC_Os01g51754 | 24.15 | 16.93 | 11.33 |
| LOC_Os07g39780 | 18.68 | 19.99 | 18.01 |

**Table S6.** Primers used in the study.

| **Name** | **Primer sequence (5’-3’)** |
| --- | --- |
| pre_miR1432_F | AGGATGTGCGTTCTTGTGGG |
| pre_miR1432_R | CAGGGTCAGTCAGTCCTATGTTC |
| pro_miR1432_F | CAATAATCTCCAATGCCCAAT |
| pro_miR1432_R | AATCTGCAGTCACATACATGCC |
| miR1432_stem_loop_F | ACACTCCAGCTGGGATCAGGAGAGATGAC |
| miR1432_stem_loop_R | CTCAACTGGTGTCGTGGAGTCGGCAATTCAGTTGAGGTCGGTGT |
| stem-loop_U | TGGTGTCGTGGAGTCG |
| miR1432 Probe | GTCGGTGTCATCTCTCCTGAT |
| PHB-ACOT_F/OXmACOT_F1 | ATGGACCCGGAAGCGGTGCG |
| PHB-ACOT_R/OXmACOT_R2 | TCATAGTTTGCTAGATACAG |
| OXmACOT_R1 | TTCCTGAAGTCTACTGATACCACGCCTACTGATTTTC |
| OXmACOT_F2 | GAAAATCAGTAGGCGTGGTATCAGTAGACTTCAGGAA |
| OsACOT_Outer | CCAGTTGCGTAATTTCTGAGGTCC |
| OsACOT_Inner | ATACAGCACTGACAGCGTATGACAAGG |
| OsACOT_F | CGAGCTCAACCTCTCCTACG |
| OsACOT_R | GCGCTATCAATTTGCCTGTC |
| OsTAA1;4_F | CTCCGGATGCTGAAGATCCT |
| OsTAA1;4_R | TCTTCCTCCCACTCGCATTT |
| OsABA4_F | CTACGCCTACCTGCTCTACC |
| OsABA4_F | GAAACCGAATGCCTGGTCTC |
| OsARF14_F | TCAGGGGATGCTGTCTTGTT |
| OsARF14_R | GAATTCTGACGCACCACTCC |
| OsPYL4_F | GAGAGACTGGAGGTTGCACT |
| OsPYL4_R | ACCGCCTCAAGAACTACCTC |
| OsSSⅡc_F | CCCATGTGAATGTTGGTT |
| OsSSⅡc_R | CTTTGCTCTTGCGGATAA |
| OsBT1-2_F | TGAGGGTGAAGATCGGGAAC |
| OsBT1-2_R | TGAAATGCTCGATGGCCTTG |
| OsPIN2_F | CAACACCTACTCCAGCCTCA |
| OsPIN2_R | GGTTGCAGAGCCATGAACAA |
| OsPIN9_F | AAGCTTCCTTGGCCTCATCT |
| OsPIN9_R | ACAACCGGGCCTATCAGAAA |
| Os01g73140_F | TCAAAGTGTTTAGCGTGCCC |
| Os01g73140_R | AACTCTATCGCGGGGAATGA |
| Os03g45720_F | CCTTCTACGAGGAGGTGCTC |
| Os03g45720_R | GTGAGGAACCCGTCGTAGTC |
| Os04g42930_F | TTTGCGTCCGTGTGAAGAAG |
| Os04g42930_R | CAGCCACCAATATGCTTCCC |
| Os04g46960.1_F | AAGCGGAAAAGACGTGAACC |
| Os04g46960.1_R | GAGTGCAAGCAAACTGGACA |
| β-actin_F | GGAAGTACAGTGTCTGGATTGGAG |
| β-actin_R | TCTTGGCTTAGCATTCTTGGGT |
